# LSD1 is required for euchromatic origin firing and replication timing

**DOI:** 10.1038/s41392-022-00927-x

**Published:** 2022-04-13

**Authors:** Yue Wang, Yunchao Huang, Edith Cheng, Xinhua Liu, Yu Zhang, Jianguo Yang, Jordan T. F. Young, Grant W. Brown, Xiaohan Yang, Yongfeng Shang

**Affiliations:** 1 Department of Biochemistry and Molecular Biology, School of Basic Medical Sciences, Peking University Health Science Center, Beijing, 100191 China; 2 Department of Biochemistry and Molecular Biology, School of Basic Medical Sciences, Hangzhou Normal University, Hangzhou, 311121 China; 3 Department of Biochemistry and Molecular Biology, School of Basic Medical Sciences, Capital Medical University, Beijing, 100069 China; 4 Department of Biochemistry and Donnelly Centre, University of Toronto, Toronto, ON M5S 1A8 Canada; 5 Lunenfeld-Tanenbaum Research Institute, Mount Sinai Hospital, 600 University Ave., Toronto, ON M5G 1×5 Canada

**Keywords:** Epigenetics, Biochemistry

## Abstract

The chromatin-based rule governing the selection and activation of replication origins remains to be elucidated. It is believed that DNA replication initiates from open chromatin domains; thus, replication origins reside in open and active chromatin. However, we report here that lysine-specific demethylase 1 (LSD1), which biochemically catalyzes H3K4me1/2 demethylation favoring chromatin condensation, interacts with the DNA replication machinery in human cells. We find that LSD1 level peaks in early S phase, when it is required for DNA replication by facilitating origin firing in euchromatic regions. Indeed, euchromatic zones enriched in H3K4me2 are the preferred sites for the pre-replicative complex (pre-RC) binding. Remarkably, LSD1 deficiency leads to a genome-wide switch of replication from early to late. We show that LSD1-engaged DNA replication is mechanistically linked to the loading of TopBP1-Interacting Checkpoint and Replication Regulator (TICRR) onto the pre-RC and subsequent recruitment of CDC45 during origin firing. Together, these results reveal an unexpected role for LSD1 in euchromatic origin firing and replication timing, highlighting the importance of epigenetic regulation in the activation of replication origins. As selective inhibitors of LSD1 are being exploited as potential cancer therapeutics, our study supports the importance of leveraging an appropriate level of LSD1 to curb the side effects of anti-LSD1 therapy.

## Introduction

DNA replication commences from multiple sites referred to as replication origins in eukaryotic cells. Despite recent advances in the understanding of origin specification, no consensus sequences with predictive values of replication origins have been found in higher eukaryotes. Moreover, only a part of replication origins is used to replicate the eukaryotic genome at each cell cycle;^1^ inactive origins are rarely used under normal conditions but can be potentially activated in specific cell programs or cellular micromilieus,^2^ pointing to epigenetic regulation in origin specification.

Replication initiation occurs in a two-step process: “licensing” and “firing”.^3^ Licensing of replication origins starts with the recruitment of the origin recognition complex (ORC1-6) and is accomplished in G_1_ phase upon cell division control protein 6 (Cdc6)- and cell division cycle 10-dependent transcript 1 (Cdt1)-mediated loading of the minichromosome maintenance (MCM) complex onto the origins, forming the pre-replicative complex (pre-RC).^4^ As cells enter S phase, origins “fire” upon transforming the pre-RC into an active DNA helicase complex through the recruitment of Topoisomerase II Binding Protein 1 (TopBP1), TopBP1-Interacting Checkpoint and Replication Regulator (TICRR, also known as Treslin), Cell Division Cycle protein 45 (CDC45), and GINS to the pre-RC.^4–6^ Replication of the mammalian genome requires sequential activation of 30,000—50,000 origins spotted with an average interval of 100 kb of DNA.^7^ In general, euchromatic regions are enriched in origins that fire early in S phase, whereas heterochromatic regions harbor origins that fire later.^8^

In recent years, evidence is accumulating to indicate that epigenetic mechanisms play important roles in the regulation of the timing and efficiency of origin firing.^9^ It is believed that alterations in epigenetic plasticity and chromatin configuration allow the generation of appropriate chromatin environments that foster the loading of ORC and/or the pre-RC, thereby promoting subsequent origin firing.^10^ Indeed, ORC is found in open chromatin that is sensitive to DNase I and is frequently associated with promoters and enhancers that are marked by active histone modifications, such as H3K27ac, H3K9ac, and H3K4me1/2.^10^ It is reported that acetylation of histones H3 and H4 accelerates origin firing or increases the efficiency of origin firing in S phase.^11^ Although H3K4me2 is implicated in direct or indirect recruitment of replication factors to origins,^12,13^ its role and regulation in mammalian DNA replication remain to be elucidated, and the chromatin-based rule that governs origin selection and activation at specific loci remains to be delineated. Moreover, lysine-specific demethylase 1 (LSD1) was characterized as an amine oxidase that catalyzes H3K4 demethylation^14^ and has ever since mainly been investigated in transcription regulation.^15,16^ Indeed, LSD1 constitutes a number of corepressor complexes including CoREST,^15,17^ CtBP,^18,19^ and NuRD.^20^ However, the role of LSD1 in DNA replication is unclear.

We report in the current study that LSD1 is physically associated with the DNA replication machinery during the DNA synthesis phase of the cell cycle. We show that LSD1 is engaged in early replication in euchromatic regions. We find that regions enriched in H3K4me2 are the preferred sites for the binding of the pre-RC, and that LSD1-catalyzed H3K4me2 demethylation is required for origin firing by promoting the binding of TICRR to the pre-RC and subsequent loading of the initiator protein CDC45. We show that LSD1 deficiency leads to a genome-wide switch from early to late in replication timing.

## Results

### LSD1 interacts with the DNA replication machinery

**Fig. 1:**
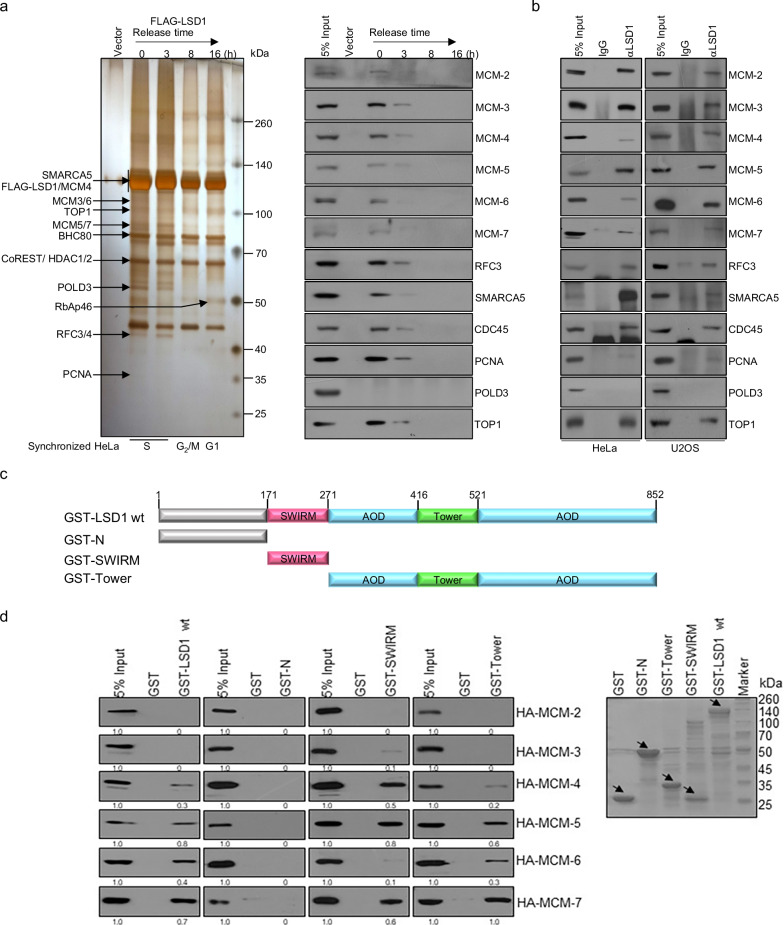
LSD1 interacts with the DNA replication machinery. **a** Immunoaffinity purification of LSD1-containing complexes. HeLa cells stably expressing FLAG-LSD1 were synchronized by double-thymidine block. Cellular extracts were immunopurified with anti-FLAG affinity columns and eluted with FLAG peptides. The eluates were resolved by SDS-PAGE and visualized by silver staining. The proteins bands were retrieved and analyzed by mass spectrometry (left). Column-bound proteins were analyzed by western blotting using antibodies against the indicated proteins (right). The percentage of input was 5%, the same for the experiments described below. **b** Immunoprecipitation of endogenous proteins in S-phase HeLa and U2OS cells with antibodies against LSD1, followed by immunoblotting with antibodies against the indicated proteins. **c** Schematic representation of the structure of LSD1. **d** GST pull-down assays with GST-LSD1, GST-N (1-171 aa), GST-SWIRM (171-271 aa), and GST-Tower (416-521 aa) and in vitro transcribed/translated MCM2-7. Coomassie brilliant blue (CBB) staining of GST-fused wild-type and deletion mutants of LSD1 is shown (arrows)

Due to its prominent role in controlling H3K4 plasticity, LSD1 has been extensively investigated in the regulation of the fundamental cellular processes such as cell proliferation and cell cycle. Indeed, LSD1 plays a critical role in cell proliferation and its deficiency results in cell cycle arrest in G_1_ and G_2_/M.^21,22^ Previously, we reported that LSD1, through its transcriptionally regulatory function, impacts cell proliferation/differentiation^23^ and influences cell reprograming.^20^ To gain more systematic understanding of the functional role of LSD1 in cell cycle regulation, we utilized an epitope-based proteomic screening with immunopurification-coupled mass spectrometry to interrogate the LSD1 interactome during the cell cycle. In these experiments, HeLa cells stably expressing FLAG-tagged LSD1 (FLAG-LSD1) were subjected to double-thymidine block, followed by release for 0, 3, 8, or 16 h to represent S (0–3 h), G_2_/M (8 h), and G_1_ (16 h) phases of the cell cycle. Cell extracts were prepared, affinity-purified using an anti-FLAG affinity gel, eluted with excess FLAG peptide, resolved on SDS-PAGE, and visualized by silver staining (Fig. 1a and Supplementary Fig. S1a). The protein bands on the gels were retrieved and analyzed by mass spectrometry. We found that LSD1 co-purified in S phase with MCM3/4/5/6/7, all components of the MCM helicase complex that is required for both initiation and elongation of DNA replication,^24^ as well as with Replication Factor C Subunit 3/4 (RFC3/4) and Proliferating Cell Nuclear Antigen (PCNA) (Fig. 1a, left). In addition, SMARCA5, one of the most conserved chromatin remodeling factors and a molecular motor for DNA replication,^25^ was also identified in the LSD1 interactome during S phase of the cell cycle. Notably, the LSD1 interactome was somewhat different during different phases of the cell cycle, at least in S phase versus G_2_/M/G_1_ phases (Fig. 1a, left), with replication proteins detected during S phase while transcription regulators like CoREST detected throughout the cell cycle. The presence of the MCM subunits and other proteins in the LSD1 interactome was confirmed by western blotting of the column-bound proteins with antibodies against the corresponding proteins (Fig. 1a, right). The detailed results of the mass spectrometric analysis are provided in Supplementary Table S1.

To confirm the interactions between LSD1 and the components of the DNA replication machinery, total proteins from HeLa or U2OS cells synchronized in S phase were extracted and co-immunoprecipitation experiments were performed with antibodies detecting the endogenous proteins. Immunoprecipitation (IP) with antibodies against LSD1 followed by immunoblotting with antibodies against MCM2, MCM3, MCM4, MCM5, MCM6, or MCM7 demonstrated that all these components of the MCM helicase complex were efficiently co-immunoprecipitated with LSD1 (Fig. 1b). Likewise, IP with antibodies against LSD1 followed by immunoblotting with antibodies against RFC3, TOP1, and SMARCA5 also showed that all these proteins were efficiently co-immunoprecipitated with LSD1 in HeLa and U2OS cells (Fig. 1b). Moreover, the DNA helicase activator CDC45 was also co-immunoprecipitated with LSD1, despite that it was not detected by mass spectrometry, whereas POLD3 (DNA polymerase δ) was not co-immunoprecipitated with LSD1 (Fig. 1b). Endogenous LSD1 IP pulldowns with chromatin fractions from S-phase HeLa cells corroborated the results (Supplementary Fig. S1b).

LSD1 harbors several distinct structural domains, including an N-terminal nuclear localization sequence ahead of a SWIRM domain plus a C-terminal Tower domain that shapes as a long helix-turn-helix protruding the catalytic AOD domain^26–28^ (Fig. 1c). The SWIRM domain is responsible for protein–protein interactions, the AOD domain binds FAD and substrates, and the Tower domain is required for both the enzymatic activity and protein interaction. To further support the interaction of LSD1 with the DNA replication machinery and to understand the molecular details involved in the interaction, glutathione S-transferase (GST) pull-down assays were performed with GST-fused LSD1 or LSD1 mutants, including N-terminal fragment (1-171 aa), the SWIRM domain (172-271 aa), or the Tower domain (417-521 aa), and in vitro transcribed/translated MCM2, MCM3, MCM4, MCM5, MCM6, or MCM7. The results showed that LSD1 interacts with MCM4/5/6/7, but not MCM2 and MCM3, through the SWIRM and Tower domain of LSD1 (Fig. 1d). Together, these results substantiate the observation that LSD1 interacts with the DNA replication machinery during S phase of cell cycle.

### The expression of LSD1 is cell cycle-regulated and peaks at S phase

**Fig. 2:**
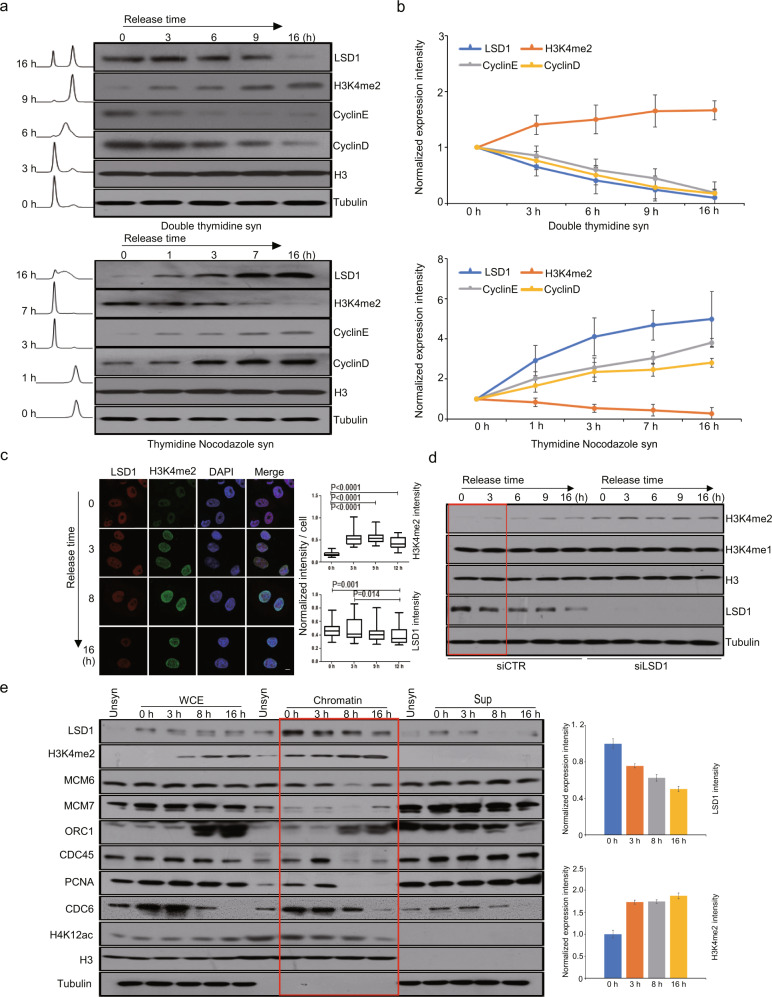
The expression of LSD1 is cell cycle-regulated and peaks at the S phase. **a** Western blotting analysis of LSD1 and H3K4me2 in double-thymidine- or thymidine-nocodazole-synchronized U2OS cells at the indicated time points after release. The traces are flow cytometry data of DNA content. **b** Quantification of the levels of LSD1 and H3K4me2 during the cell cycle. Each point represents the mean ± SD for three independent experiments. **c** Immunostaining of LSD1 and H3K4me2 in double-thymidine-synchronized U2OS cells at the indicated time points after release (left). Quantification of immunostaining intensity of LSD1 and H3K4me2 during the cell cycle (right). *P* values were calculated by a two-tailed student’s *t*-test. Scale bar, 10 μm. **d** U2OS cells were transfected with control siRNAs or LSD1 siRNAs for western blotting analysis of H3K4me2/1 during the cell cycle progression. **e** U2OS cells were synchronized by double-thymidine block prior to release for western blotting analysis of chromatin-bound LSD1 at early S (0 h), S (3 h), G_2_ (9 h), or G_1_ phase (16 h) of the cell cycle. ORC1, MCM6, MCM7, and CDC6 are components of the pre-RC. CDC45 and PCNA are factors representing active replication. Quantification of the levels of LSD1 and H3K4me2 during the cell cycle (right). Each point represents the mean ± SD for three independent experiments

To explore the role of LSD1 in DNA replication, we next profiled the expression pattern of LSD1 during the cell cycle. To this end, U2OS cells were synchronized at G_1_/S phase with double-thymidine or at G_2_/M phase with thymidine-nocodazole. The expression of LSD1 was analyzed by western blotting at different times after release. The results showed that LSD1 was expressed throughout the cell cycle (Fig. 2a). Strikingly, despite the current understanding that LSD1 mainly functions in transcription regulation, which occurs primarily in G_1_ phase, the peak of LSD1 expression was detected at S phase (Fig. 2a). Quantification of three independent experiments showed that LSD1 was highly expressed in the early stage of S phase, and its level gradually decreased as cells exited from S phase (Fig. 2b), suggesting a functional involvement of LSD1 in early S phase. Consistent with this notion, the level of LSD1 expression was inversely correlated with the level of H3K4me2 on bulk chromatin during the cell cycle (Fig. 2a, b).

Immunofluorescent staining was then performed to detect LSD1 in U2OS cells during the cell cycle. In these experiments, U2OS cells were synchronized in G_1_/S by double-thymidine block. After release at different times, cells were fixed and immunostained with antibodies against LSD1 and H3K4me2. While LSD1 signal was detected throughout the cell cycle, its intensity peaked in S phase (0 and 3 h) (Fig. 2c). Correspondingly, the signal of H3K4me2 decreased at the beginning of S phase (0 h) and re-established as cells progressed through S phase (Fig. 2c). Although H3K4me2 could be passively diluted during replication by incorporation of newly synthesized histone H3,^29^ the extent of the H3K4me2 reduction in the very beginning of S phase could not be simply explained by dilution. In addition, knockdown of LSD1 in U2OS cells resulted in an increase in H3K4me2 level on bulk chromatin (Fig. 2d). Indeed, LSD1 was present in the chromatin fraction in G_1_/S-synchronized U2OS cells progressing through S phase to mitosis, with a higher level of chromatin-bound LSD1 detected in S phase (Fig. 2e). Correspondingly, H3K4me2 enrichment reduced in early S phase and restored as S phase progressed. In addition, the level of H4K12 acetylation changing from high to low indicates the maturation of newly deposited histone H4 proteins, which are acetylated at K12 and mature through deacetylation as S phase progressed (Fig. 2e). Together, these data indicate that LSD1 accumulates on chromatin during S phase and its level is inversely correlated to that of H3K4me2.

### LSD1 is required for efficient DNA replication

**Fig. 3:**
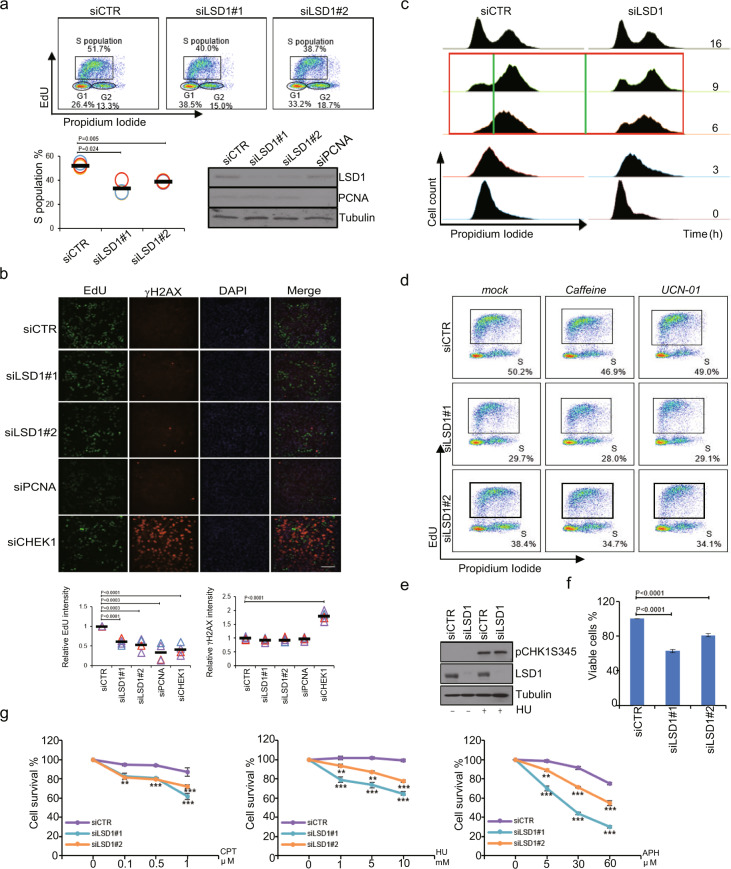
LSD1 is required for DNA replication. **a** LSD1-deficient U2OS cells were subjected to EdU incorporation analysis. The percentage of cells in each phase of the cell cycle was determined by dual PI/EdU flow cytometry (top), and the percentage of S-phase cells is plotted. Each bar represents mean ± SD for three independent experiments. The efficiency of knockdown was verified by western blotting with PCNA as a control. **b** LSD1-deficient U2OS cells were subjected to immunofluorescent staining for EdU and γH2AX. Cells depleted of PCNA and CHEK1 were used as positive controls for replication defects and spontaneous DNA damage, respectively, and the immunofluorescent signal of EdU and γH2AX was quantitated. Each bar represents mean ± SD for three independent experiments. Scale bar, 200 μm. **c** LSD1-deficient U2OS cells were synchronized at the G_1_/S transition by double-thymidine block, released for different times, and analyzed for DNA content by flow cytometry. **d** LSD1-deficient U2OS cells were treated with caffeine or UCN-01 for EdU incorporation analysis. **e** Western blotting analysis of pCHK1S345 in HeLa cells depleted of LSD1. **f** LSD1-deficient U2OS cells were subjected to cell viability assays. Each bar represents the mean ± SD for three independent experiments. **g** LSD1-deficient U2OS cells were challenged with different doses of CPT, HU, or APH for cell viability assays. Each point represents the mean ± SD for three independent experiments (**p < 0.01, ***p < 0.001; *t*-test)

S phase mainly experiences DNA duplication.^30^ The accumulation of LSD1 in S phase supports a role for LSD1 in DNA replication. To test this, U2OS cells were treated with control siRNAs or siRNAs against LSD1 and pulse-labeled with EdU. Cells were then fixed and analyzed by flow cytometry after EdU conjugation to Alexa Fluor 647 and propidium iodide (PI) staining. Knockdown of LSD1 was associated with a decreased number of cells in S phase (Fig. 3a and Supplementary Fig. S1c), suggesting a role for LSD1 in either defective S-phase progression, G_1_ arrest or accelerated progression through S phase. Meanwhile, pulse-labeling of LSD1-depleted U2OS cells with EdU followed by Alexa Fluor 488 conjugation showed that the nuclear EdU fluorescent intensity, which represents newly synthesized DNA in nuclei, markedly decreased (Fig. 3b), supporting a role for LSD1 in DNA synthesis. The fluorescent intensity of EdU and γH2AX was quantified using ~1500 cells for each sample (Fig. 3b, bottom). PCNA and CHEK1 served as positive controls in staining of EdU and γH2AX, respectively.

To investigate the effect of LSD1 on S-phase progression, LSD1-depleted U2OS cells were synchronized at G_1_/S transition by double-thymidine block followed by release for 0, 3, 6, 9, or 16 h. Analysis of DNA content showed that S-phase progression was impeded upon knockdown of LSD1 (Fig. 3c and Supplementary Fig. S1d). Defects in S-phase progression might be due to activation of DNA damage response, specifically the activation of S-phase checkpoint by phosphatidyl inositol 3’ kinase-related kinases ATM and ATR.^31^ Checkpoint activation would result in a transient cell cycle arrest during S phase, allowing cells to repair the damage, complete DNA replication, and proceed to mitosis.^32^ However, immunostaining showed that the level of γH2AX was comparable in control cells and LSD1-deficient cells (Fig. 3b), arguing against the possibility that the decreased incorporation of EdU in LSD1-deficient cells was due to replication stress-inflicted DNA damage response. To substantiate this, LSD1 siRNAs-treated U2OS cells were exposed to caffeine, an ATM and ATR inhibitor,^33^ or UCN-01, a CHK1 inhibitor,^34^ and pulse-labeled with EdU for 1 h. PI/EdU incorporation analysis by flow cytometry showed that neither caffeine nor UCN-01 could restore S-phase progression in LSD1-silenced cells (Fig. 3d and Supplementary Figs. S1e and S2a). Moreover, western blotting with anti-phosphor-Chk1ser345 detected no CHK1 activation (Fig. 3e). Together, these results indicate that knockdown of LSD1 was associated with a checkpoint-independent blockage of S-phase progression.

DNA replication is intrinsically linked to cell proliferation and viability, especially in response to replication stresses, e.g., treatment with hydroxyurea (HU) or camptothecin (CPT).^35^ To further support the role of LSD1 in DNA replication, U2OS cells were transfected with control siRNAs or LSD1 siRNAs, and cell proliferation was measured by cell viability assays. LSD1 knockdown resulted in a mild yet significant decrease in cell numbers (Fig. 3f). Moreover, challenging U2OS cells with an increasing concentration of HU, CPT, or Aphidicolin showed that LSD1 deficiency exacerbated the decrease in cell viability inflicted by these DNA-damaging agents (Fig. 3g).

Next, LSD1-depleted HeLa cells were labeled with BrdU and stained for pRPA32S33 to test if LSD1 deficiency is associated with stalled replication forks. No differences in pRPA32S33 staining were detected in control versus LSD1-deficient cells (Supplementary Fig. S2b). In addition, LSD1-deficient cells treated with HU, CPT, or Aphidicolin were also stained for pRPA32S33 and BrdU. Although the intensity of the fluorescent signals of pRPA32S33 increased under the treatment of HU, CPT or Aphidicolin, no significant differences were observed in control versus LSD1-deficient cells (Supplementary Fig. S3a). Together, these results argue against a scenario that LSD1 depletion is associated with stalled replication forks.

### LSD1 is associated with replication forks

**Fig. 4:**
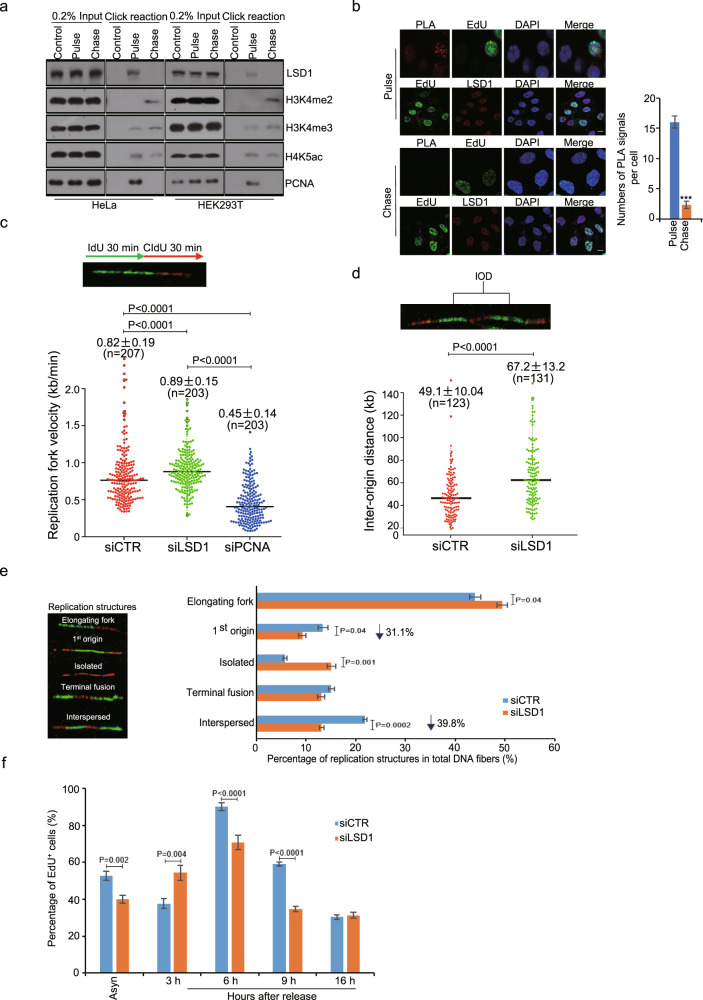
LSD1 deficiency compromises replication initiation. **a** iPOND assays for LSD1 at replication forks or mature chromatin in HeLa and HEK293T cells. Pulldowns of control, nascent DNA (pulse), or mature chromatin (chase) were analyzed by western blotting with the indicated antibodies. **b** U2OS cells were pulse-labeled with EdU or followed by thymidine chase for in situ PLA assays. The efficacy of the antibodies was determined for each assay. PLA, red; biotin, green; DAPI, blue. Scale bar, 10 μm. Quantification of the PLA signals is shown on the right (*n* = 15 cells). **c** LSD1-deficient U2OS cells were pulse-labeled successively with IdU and CldU for DNA fiber assays. Distribution of replication forks is presented as beeswarm-box plots (*n* ≥ 200). The median fork rate for each experiment is shown. **d** IOD was measured on spread DNA fibers and presented as beeswarm-box plots (*n* ≥ 100). **e** Five classes of replication structures were defined (*n* ≥ 200). Left, representative classes of replication structures. Right, the percentage of the different classes is scored. **f** Synchronized U2OS cells were analyzed at different time points of release for EdU incorporation by flow cytometry. *P*-values are indicated

To further explore the role of LSD1 in DNA replication, Isolation of Proteins On Nascent DNA (iPOND) methodology^36^ was utilized and the spatiotemporal distribution of LSD1 at replication forks and re-assembled chromatin was analyzed. In these experiments, nascent DNA in proliferating HeLa or HEK293T cells was labeled with EdU for 10 min followed by thymidine chase for 30 min to allow chromatin re-assembly and fork movement away from EdU label.^37^ Biotin conjugation to EdU-labeled DNAs by click chemistry facilitates a single-step streptavidin purification of the proteins bound to nascent (EdU labeling only) and mature (EdU labeling followed by thymidine chase) DNAs. Western blotting showed that LSD1 was associated with nascent but not mature DNA (Fig. 4a), suggesting that LSD1 is associated with DNA replication forks during DNA replication, but not regions distal to DNA replication forks. Consistently, H3K4me2 was more enriched in mature chromatin than in nascent DNAs (Fig. 4a).

To reinforce the observation that LSD1 is associated with DNA replication forks, in situ proximity ligation assays (PLA)^38,39^ were performed to test if LSD1 and nascent DNAs were in a close proximity. To this end, U2OS cells were labeled with EdU for 10 min followed by a thymidine chase for 30 min. LSD1 and EdU-biotin were detected with corresponding antibodies followed by secondary antibodies linked to oligonucleotides that form a closed circle upon addition and ligation of complementary linkers. Fluorescent signals are produced by amplifying rolling circle in the presence of fluorescence-labeled oligonucleotides. Confocal microscopy showed that while PLA signals were readily detected in cells labeled with EdU, such signals were barely detected in thymidine-chased samples (Fig. 4b). The proportion of cells with LSD1-EdU PLA signals among EdU^+^ cells was up to ~30% (Supplementary Fig. S3b). These observations support the proposition that LSD1 is involved in initiation rather than elongation of DNA replication.

### LSD1 is involved in the initiation of DNA replication

To further differentiate the role of LSD1 in initiation or elongation of replication, replication was assessed in LSD1-deficient cells by DNA fiber assays.^40^ In these experiments, LSD1-depleted asynchronous U2OS cells were pulse-labeled consecutively with 5’-iododeoxyuridine (IdU) and 5’-chlorodeoxyuridine (CldU). DNA fibers were then spread onto salinized glass slides, and the rate of replication fork progression was calculated by the length of IdU tracks as a function of time. We found that the length of IdU tracks in LSD1-depleted cells was longer than that in control cells (Fig. 4c and Supplementary Fig. S4a), suggesting an accelerated fork progression. Analysis of the distribution of active origins on DNA fibers revealed that the inter-origin distance increased by 37% in cells deficient in LSD1 (Fig. 4d and Supplementary Fig. S4b). This is consistent with a previous report that deficiency of Orc1 or Cdc7 was associated with a decreased origin firing and accelerated fork velocity,^41^ favoring a role for LSD1 in the initiation of DNA replication. Meanwhile, analysis of the fiber data revealed that first origin and interspersed structures, represented by the fraction of origins labeled with IdU, decreased in LSD1-deficient cells, whereas isolated structures, which were labeled with CIdU only and represent second origins, increased (Fig. 4e), suggesting a possibly compensatory activation of dormant origins upon depletion of LSD1. Intriguingly, FACS analysis of cell cycle progression indicated an increased EdU incorporation at the early stage of S phase (3 h) and reduced EdU incorporation at middle/late S phase (6 h) in LSD1-deficient cells (Fig. 4f). Together with the results of the fiber assays, these observations support the notion that the velocity of the replication fork and inter-origin distance increased in cells deficient of LSD1. The increased velocity of the replication fork could be a functional adaption to inefficient replication initiation that might account for the increased EdU incorporation in early S phase, but the overall S progression was compromised by LSD1 knockdown (Fig. 3c and Supplementary Fig. S1d).

### LSD1 regulates genome-wide replication timing

**Fig. 5:**
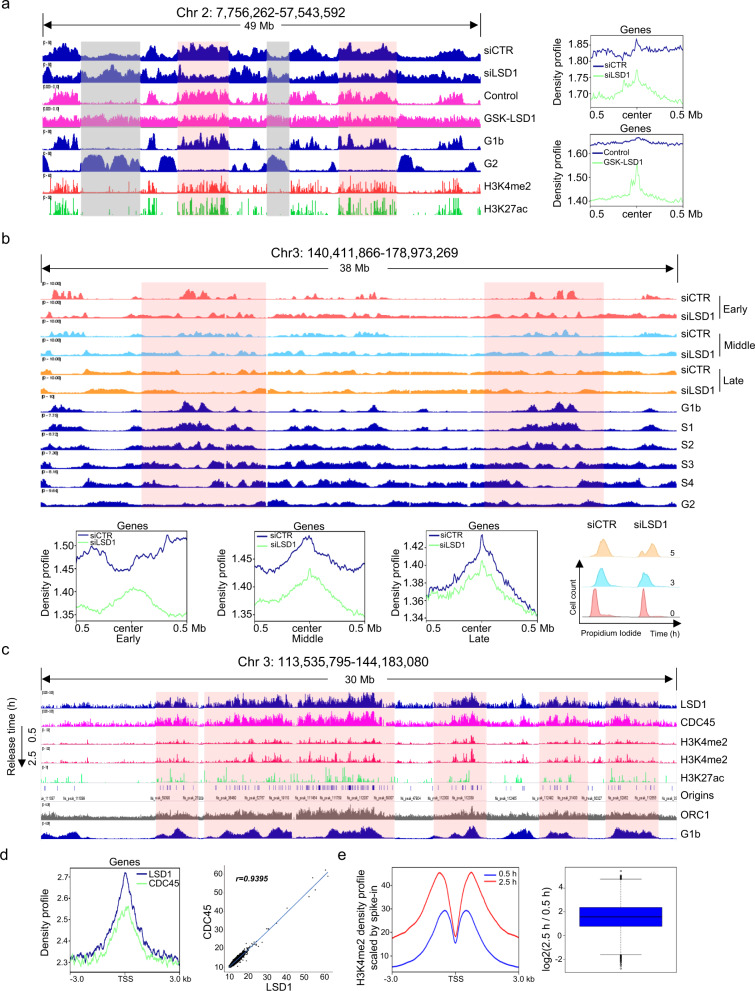
LSD1 regulates genome-wide replication timing. **a** Comparison of genome-wide replication timing after LSD1 was depleted or inhibited. HeLa cells were transfected with LSD1 siRNAs or treated with GSK-LSD1 prior to synchronization in early S phase for Repli-seq. The results from a representative region of chromosome 2 spanning 49 Mb are illustrated. Regions with decreased Repli-seq reads density after LSD1 depletion/inhibition are shaded in pink, and regions with increased reads density shaded in gray. Replication profiles from public data representing early (G1b) and late (G2) S phases and the distribution of H3K4me2 and H3K27ac are shown. Numbers in square brackets indicate the data range of corresponding track. Statistical analysis of Repli-seq reads density around the summits of LSD1 binding sites is shown (right panel). **b** Different time points of synchronized LSD1-depleted HeLa cells were analyzed by Repli-seq. Replication profiles from public data representing early (G1b and S1), middle (S2 and S3), and late (S4 and G2) S phases (dark blue) are shown. Statistical analysis of Repli-seq reads density around the summits of LSD1 is shown (lower panel). Full cell cycle plots of LSD1-deficient HeLa cells for Repli-seq (right). **c** HeLa cells were synchronized via double-thymidine block followed by release for 0.5 or 2.5 h to enrich cells in early S phase. Genomic distributions of LSD1, CDC45, and H3K4me2 were determined by ChIP-seq analysis. A representative region spanning 30 Mb in chromosome 3 is shown. Origins and ORC1 ChIP-seq data in HeLa cells were from published data. Public Repli-seq data of early S phase (G1b) is shown below. Co-occurrence of LSD1, CDC45 and H3K4me2 on early-replication regions are shaded. **d** Whole-genome analysis of the distribution of LSD1 and CDC45. *r*, Pearson’s correlation coefficient. **e** The changes in H3K4me2 were quantitated by ChIP-seq with exogenous *Drosophila melanogaster* genome as a reference. Scale factors for 0.5 and 2.5 h are 24.8 and 11, respectively (left). Log2-based ratio of H3K4me2 signal density at 2.5 and 0.5 h of 3000-bp up- and down-stream of TSS of all genes are indicated (right)

To investigate the effect of LSD1 on replication at a genome-wide level, Repli-seq^42,43^ was performed to identify replicated regions during S phase following LSD1 depletion. HeLa cells were used for these experiments as Repli-seq and histone modification datasets from HeLa cell line are available from the Encyclopedia of DNA Elements (ENCODE) project.^44^ HeLa cells were treated with control or LSD1 siRNAs, followed by synchronization with double-thymidine block prior to release for 2 h to accumulate cells in early S phase. Cells were then pulse-labeled with EdU for 10 min, fixed, and permeabilized.^45^ Incorporated EdU was conjugated to biotin, chromatin was sheared, and replicated DNAs were isolated by incubating with streptavidin magnetic beads prior to sequencing. The sequence reads were mapped to the reference genome (GRCh38/hg38) and reads intensity was visualized in Integrative Genome Viewer (IGV) (Fig. 5a). The data were cross-analyzed with public Repli-seq data representing early (G1b) or late (G2) S phase.^42^ LSD1 depletion led to a substantially reduced Repli-seq reads density in early initiation zones which generally replicated in G1b and recognized as enhancer-like euchromatin enriched in H3K4me2 and H3K27ac,^46,47^ suggesting that LSD1 deficiency results in a compromised replication in these early initiated regions (Fig. 5a and Supplementary Fig. S5a). In contrast, Repli-seq reads density from the adjacent zones devoid of these two histone marks, which represent passively replicated regions or late-initiated regions that are generally replicated in G_2_, increased (Fig. 5a). These observations point to a role for LSD1 in facilitating replication initiation in enhancer-like euchromatin in early S phase and in regulating replication timing.

To address the question that whether or not the enzymatic activity of LSD1 is required for its function in DNA replication, Repli-seq was performed in HeLa cells under the treatment of the LSD1 inhibitor GSK-LSD1.^48^ In line with LSD1 depletion, GSK-LSD1 treatment led to a reduced Repli-seq reads density in early initiation zones and an increased reads density in the adjacent zones or late-initiated regions (Fig. 5a, left panel), indicating that the enzymatic activity of LSD1 is required for its function in DNA replication. Consistently, statistical analysis of the Repli-seq results showed that the reads density around LSD1 summits decreased in LSD1 knockdown and GSK-LSD1 groups (Fig. 5b, right panel). Collectively, these observations support the arguments that LSD1-engaged DNA replication occurs in enhancer-like euchromatin and early replication regions, and that LSD1 depletion led to a genome-wide shift from early to late in replication timing.

Repli-seq was also performed in LSD1-depleted HeLa cells that were synchronized to G_1_/S phase by thymidine/aphidicolin double treatments prior to release for 0, 3, and 5 h to accumulate cells in early, middle and late S phase (Fig. 5b and Supplementary Fig. S5b). Cells were then pulse-labeled with EdU for 10 min before DNA extraction.^45^ The Repli-seq results from control groups were consistent with that from the published Repli-seq dataset (GSE37583) (Supplementary Fig. S6a). In line with Fig. 5a, LSD1 knockdown led to an increased reads density from the late-initiated regions in early S phase (0 h), supporting a reversed replication timing. With the progression of S phase (3 and 5 h), the reads density in the early initiation zones was still low, suggesting that these regions were still waiting to be passively replicated in the end of S phase. Consistently, statistical analysis showed that the reads density around LSD1 summits decreased in LSD1 knockdown groups (Fig. 5b, lower panel), indicating that LSD1-engaged DNA replication and the overall S progression were impeded by LSD1 knockdown even compensatory mechanisms of replication existed.

To gain further support of the involvement of LSD1 in euchromatin replication and to investigate the genomic landscape of LSD1 binding, we performed chromatin immunoprecipitation coupled with deep sequencing (ChIP-seq). To this end, HeLa cells were synchronized via double-thymidine block prior to release for 0.5 or 2.5 h to isolate cells in early S phase. Soluble chromatin was prepared and ChIP-seq was performed using antibodies against LSD1, CDC45, or H3K4me2. The data were then cross-analyzed with public G1b Repli-seq and the origin data from HeLa cells profiled in a recent study.^49^ We found that at early S phase (0.5 h), both LSD1 and CDC45 binding distributed in a broad region, represented by a region spanning 30 Mb on chromosome 3. Importantly, the distribution pattern of LSD1 largely coincided with that of CDC45 on the early-replicated regions in enhancer-like elements marked with H3K4me2 and H3K27ac (Fig. 5c and Supplementary Fig. S6b). Cross-analysis with the published ORC1 ChIP-seq data^49,50^ showed that the genomic distribution of CDC45 and LSD1 only partially overlapped with that of ORC1 (Fig. 5c and Supplementary Fig. S6b), consistent with the notion that not all ORC binding sites undergo replication initiation in a given cell cycle.^51^ Remarkedly, the genome-wide distribution of H3K4me2 significantly decreased at the onset of S phase (0.5 h) and re-established quickly with the progression of DNA replication (2.5 h) (Fig. 5c), supporting a notion that this modification is highly dynamic and needs to be erased temporally to facilitate the firing of early origins. In addition, genome-wide analysis indicated that LSD1 and CDC45 were co-enriched across whole genome (Fig. 5d). Furthermore, ChIP-seq was performed with antibodies against H3K4me2 using exogenous genome (ChIP-Rx)^52^ as a reference in HeLa cells synchronized by double-thymidine block prior to release for 0.5 or 2.5 h. We aligned the sequence reads to the mixed reference genome containing GRCh38/hg38 and DM6 and normalized human H3K4me2 intensity signals with the *Drosophila* signals. Comparison of the normalized density profile between 0.5 and 2.5 h surrounding the genome-wide transcriptional start sites revealed that the genome-wide distribution of H3K4me2 was significantly low at the beginning of S phase (0.5 h) (Fig. 5e). Together, these results highlight the importance of LSD1 in replication initiation of the enhancer-like euchromatic regions.

### LSD1 promotes origin firing by facilitating TICRR recruitment and CDC45 loading

**Fig. 6:**
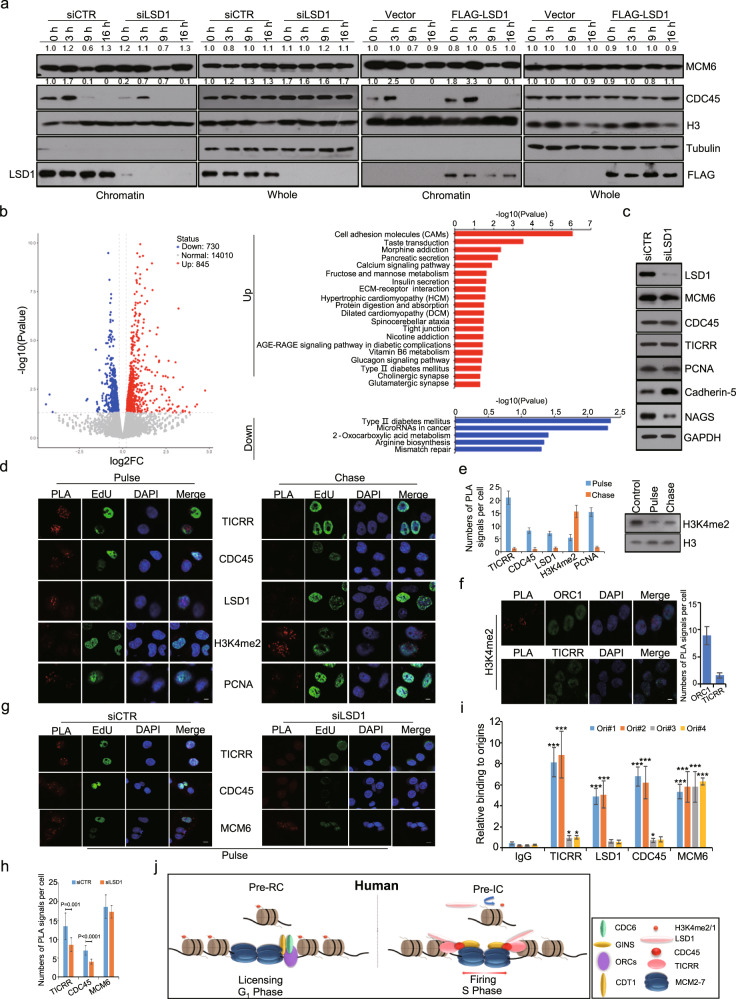
LSD1 promotes origin firing through H3K4me2 demethylation-dependent loading of TICRR. **a** LSD1-deficient or overexpressing U2OS cells were synchronized by double-thymidine block followed by release for 0 and 3 (S), 9 (M), and 16 (G_1_) h. Chromatin fractions were analyzed by western blotting for MCM6, CDC45, and PCNA. The bands were quantified using ImageJ software. **b** Genome-wide transcriptome analysis by RNA sequencing in LSD1-deficient U2OS cells. The differentially expressed genes are shown in volcano graphs (left) and analyzed by KEGG database for the significant pathway (right). **c** Western blotting analysis of MCM6, TICRR, CDC45, and PCNA in U2OS cells depleted of LSD1. Cadherin-5 and NAGS were used as positive or negative control. **d** PLA assays for the proximity of TICRR, CDC45, LSD1, or H3K4me2 relative to EdU-biotin-labeled nascent DNAs in U2OS cells following immunostaining for EdU-biotin (green). PCNA was used as a positive control. Left, cells pulse-labeled with EdU. Right, cells pulse-labeled with EdU and chased with thymidine. Scale bar, 10 μm. **e** Quantification of PLA spots (*n* = 8 cells) (left). Western blot of H3K4me2 following the thymidine chase (right). **f** PLA assays for the proximity of ORC1 or TICRR relative to H3K4me2 followed by immunostaining for ORC1 or TICRR (green). Quantification of the PLA signals is shown right (*n* = 12 cells). Scale bar, 10 μm. **g**, **h** PLA assays in LSD1-deficient U2OS cells for the proximity of TICRR, CDC45, or MCM6 relative to biotin-labeled nascent DNAs followed by immunostaining for biotin (green). Scale bar, 10 μm. Quantification of PLA spots from three independent experiments is shown below (*n* = 22 cells). *P-*values were determined by two-tailed student’s *t*-test. **i** qChIP assays for TICRR, CDC45, LSD1, and MCM6 at early origins (Ori#1 and 2) and late origins (Ori#3 and 4). Results are presented as fold of change over input with IgG as a negative control. Each bar represents mean ± SD for three independent experiments (*p < 0.05, **p < 0.01, ***p < 0.001; *t*-test). **j** The proposed model of LSD1-regulated DNA replication initiation in human cells

Defective replication initiation could be a result of defective licensing or failed origin firing. To distinguish these, the loading of MCM6 (representing licensing) and CDC45 (representing firing) on chromatin upon LSD1 depletion or overexpression was analyzed by chromatin fractionation assays. Neither knockdown nor overexpression of LSD1 had an evident effect on the loading of MCM6 onto S-phase chromatin (Fig. 6a, 0 and 3 h). However, LSD1 knockdown was associated with a reduction of CDC45 loading and LSD1 overexpression was accompanied by an increase in CDC45 loading onto S-phase chromatin (Fig. 6a, 0 h and 3 h). These results disfavor the engagement of LSD1 in “licensing” rather support a role for LSD1 in origin firing by promoting CDC45 loading onto the pre-RC in the early stage of DNA replication.

To exclude the possibility that LSD1 depletion affects the expression of genes involved in DNA replication, genome-wide transcriptome analysis was performed by RNA sequencing (RNA-seq). Total RNAs were prepared from U2OS cells transfected with control siRNAs or LSD1 siRNAs for RNA-seq. While 845 genes were upregulated and 730 genes were downregulated upon LSD1 knockdown (Fig. 6b), the expression of the genes that are involved in DNA replication had no significant changes upon LSD1 depletion (Fig. 6c). We confirmed the RNA-seq data by western blotting using antibodies against MCM6, TICRR, CDC45, and PCNA, with Cadherin-5 and NAGS as positive controls, whose expression was up-regulated or down-regulated, respectively, upon LSD1 depletion (Fig. 6c).

Incorporation of CDC45 into the pre-RC is a hallmark of origin firing.^53^ Thus, the observation that LSD1 is required for CDC45 loading onto the pre-RC is an indication that LSD1 is involved in the transformation of pre-RCs into the short-lived pre-initiation complex. The loading of CDC45 onto chromatin relies on TICRR,^5^ whose recruitment to origins is regulated by phosphorylation and other epigenetic cues.^6,54^ To test whether or not LSD1 influences the recruitment of TICRR, we determined if LSD1-catalyzed demethylation of H3K4me2 influences the binding of TICRR to chromatin. To this end, U2OS cells were labeled with EdU for 10 min followed by a thymidine chase for 30 min. PLA experiments detected the association of TICRR or CDC45 with nascent DNAs in EdU-labeled cells (Fig. 6d), whereas the PLA signals reflecting the association between H3K4me2 and nascent DNAs in EdU-labeled cells were much weaker than that in chased cells (Fig. 6d, e), indicating that H3K4me2 was erased temporarily during the onset of DNA replication. In addition, PLA assays in U2OS cells showed that while PLA signals between ORC1 and H3K4me2 were readily detected, PLA signals between TICRR and H3K4me2 was rarely seen (Fig. 6f). Moreover, loss-of-function experiments in U2OS cells showed that LSD1 deficiency led to a significant decrease in the number of PLA signals between TICRR/CDC45 and nascent DNAs, whereas PLA signals indicative of MCM6-nascent DNAs interaction were not affected (Fig. 6g, h). Collectively, these results support a model in which LSD1-catalyzed demethylation of H3K4me2 facilitates the recruitment of TICRR and the chromatin loading of CDC45, thereby promoting origin firing. qChIP assays with antibodies against LSD1, TICRR, MCM6, and H3K4me2 on early origins (Ori#1 and #2) and late origins (Ori#3 and #4) selected from published data^49^ showed that LSD1, CDC45, and TICRR were co-enriched only on early origins, whereas MCM6 existed on both early and late origins (Fig. 6i).

## Discussion

DNA replication initiates in broad zones in mammalian cells.^55^ These regions are mostly transcriptional inactive, often but not always flanked by active genes, and generally reside in open chromatin and enhancers marked with H3K4me1/2 and H3K27ac.^56^ Replication timing is tightly regulated, with most origins firing early in S phase, fewer later and only a small subset in the final stage of the S phase.^57^ Although the molecular events that lead to the initiation of DNA replication have been extensively studied, outstanding questions remain concerning the location of origins and the determinants that govern the activation of origins. We propose that ORC1 binding sites are marked by H3K4me2, which is erased by LSD1 to encourage the recruitment of TICRR and subsequent loading of CDC45, thereby promoting the transition of the pre-RC into active DNA helicases to fire origins. Evidence is accumulating to suggest that histone modifications play key roles in regulating the origin function and replication process.^58^ A previous study using yeast described a role for H3K4me2 in origin function.^12^ In addition, it was found that specific histone modifiers are recruited to the replication machinery at replication origins,^58^ and that H3K4me3 demethylase JARID1C is also involved in early origin firing.^34^ It is now clear that epigenetic factors are required for the function of replication origins, in which epigenetic modifications foster appropriate chromatin environments, allowing ORC binding and subsequent origin firing.

LSD1 has been extensively studied for its transcriptional regulation.^20^ In the current study, we reported that LSD1 interacts with the DNA replication machinery, and that LSD1 deficiency causes defective replication initiation and a genome-wide early-to-late shift of replication timing. Defective replication initiation may be due to defective licensing or unsuccessful origin firing. Interestingly, either loss-of-function or gain-of-function of LSD1 has little influence on the chromatin loading of the pre-RC components such as MCM6 during G_1_ phase, whereas the loading of CDC45, an indicator of DNA replication initiation, decreased in LSD1-depleted cells and increased in LSD1-overexpressing cells in early S phase, suggesting a critical role of LSD1 in origin firing. Cell cycle analysis showed that the whole replication process was compromised in LSD1-depleted cells with a significant increase of EdU incorporation at early S phase and reduction of EdU incorporation at middle and late S phase. According to our Repli-seq results, defects in early origin firing might lead to activation of dormant or late origins in adjacent regions. Thus, the acceleration of EdU incorporation at early S phase may be a result of a compensatory effect in response to defects in early origin firing. Although we cannot completely exclude a secondary effect of LSD1 on gene expression, the results of our RNA-seq support a direct role of LSD1 in replication.

We showed that H3K4me2 favors the formation of the pre-RC complex, consistent with a previous study suggesting a coincidence between licensing and H3K4me2.^34^ Nevertheless, our ChIP-seq assays showed that the enrichment of H3K4me2 across the whole genome decreased at the onset of S phase, supporting our proposition that H3K4me2 is erased at the onset of S phase to facilitate the binding of TICRR and CDC45 to the pre-RC to drive the transition from licensing to firing. Our observations are consistent with a recent report indicating that LSD1 is in a proximity with nascent DNA.^59^ Our results are also in agreement with a role of H3K4 methylation by MLL in preventing CDC45 binding to origins during checkpoint activation.^60^ In addition, a previous study showed that JARID1C facilitates early origin firing by demethylating H3K4me3,^34^ as stated above, and a more recent study in yeast showed a progressive loss of H3K4me3 during S phase on replicating regions.^61^ Given that H3K4me3 is enriched at active promoters, while H3K4me1/2 is enriched at enhancers,^47^ it is possible that both JARID1C and LSD1 contribute to euchromatic origin firing, with the former acting in promoters and the latter in enhancer-like regions. Of note, LSD1 catalyzes H3K4me1/2 demethylation favoring chromatin condensation. Indeed, studies by ourselves^62^ and the others^63,64^ reported that chromatin experiences a transient compaction in the early stage of replication, possibly functioning to clean off transcription factors. Clearly, the chromatin dynamics associated with replication initiation need further investigations. Nevertheless, a transient chromatin compaction in the early stage of replication is consistent with our observation that LSD1 is engaged in replication initiation.

Replication timing is dictated, at least in part, by chromatin features that encourage the access of initiation factors to replication origins.^65^ In mammalian cells, the level of CDC45 is low relative to licensed origins, allowing only a subset of licensed origins to fire upon S-phase entry.^66^ It is possible that in LSD1-depleted cell, firing factors such as TICRR and CDC45 are not effectively loaded onto early replication origins, leaving late origins access to these factors. Our results suggest a critical role for LSD1 in defining subset(s) of origins for early initiation and enhancer-like regions to fire when cells enter S phase.

LSD1 is highly expressed in different types of cancer, including bladder cancer, colorectal cancers, estrogen receptor-negative breast cancer, and prostate cancer.^67–69^ Consequently, selective inhibitors of LSD1 have been exploited as potential therapeutics for various cancers while with worrisome side effects.^70,71^ Gene ablation studies revealed a complex biology for this histone demethylase, probably due to the engagement of LSD1 in multiple biological processes. We report in the current study an unappreciated role for LSD1 in replication of euchromatic origin, adding to the understanding of not only origin selection, but also the pathophysiological function of LSD1 and anti-LSD1 therapeutic pursuits.

## Materials and methods

### Reagents and antibodies

Click-iT^®^ EdU Flow Cytometry Assay Kit (Cat.# C10419), Alexa Fluor^®^ 488 azide (Cat.# A10266), and biotin-azide (Cat.# B10184) were from Invitrogen. Primary antibodies against: FITC (Alexa Fluor-488, Cat.# A21202) from Molecular Probes; FLAG (M2 clone, anti-mouse, 1:1000), TICRR (HPA055024), and Tubulin (Cat.# T5168) from Sigma-Aldrich; LSD1 (Cat.# ab17721), MCM2 (Cat.# ab4461), MCM3 (Cat.# ab4460), MCM4 (Cat.# ab154315), MCM5 (Cat.# ab17967), MCM6 (Cat.# ab201683), MCM7 (Cat.# ab179904), ORC1 (Cat.# ab85830), DNA Polymerase delta (Cat.# ab186407), SMARCA5 (Cat.# ab3749), RFC3 (Cat.# ab15340), and histone H3 (Cat.# ab1791) from Abcam; CDC45 (Protein Tech Group, Cat.# 15678-1-AP); γH2AX (Cat.# 2577S) and cyclin E (Cat.# 4129) from CST; H3K4me2 (Cat.# 39679), H3K4me2 (Cat.# 39142), and H3K4me1 (Cat.# 39297) from Active motif; and PCNA (Santa Cruz, Cat.# sc-56); Secondary antibodies: HRP-conjugated mouse and rabbit IgG from GE Healthcare; ECL Detection System from Amersham; Cell viability analysis was performed with alamarBlue^®^ reagents (Thermo Fisher, Cat.# DAL1025, 1:10 dilution); Duolink in situ detection Reagent Red (Cat.# DUO92008), Duolink in situ PLA probe anti-rabbit minus (Cat.# DUO92005), and Duolink in situ PLA probe anti-mouse plus (Cat.# DUO92001) from Sigma-Aldrich; Biotin (Jackson ImmunoResearch, Cat. #200-002-211); GSK-LSD1 (MCE, HY-100546A).

### Cell culture

HeLa, U2OS, and HEK293T cells were cultured in Dulbecco’s modified Eagle’s medium (Gibco) supplemented with 10% fetal bovine serum (Gibco) and penicillin–streptomycin antibiotics (Sigma). siRNAs and control siRNAs were from GE Dharmacon, and the sequences are: siLSD1 #1: UGAAUUAGCUGAAACACAA, siLSD1 #2: GACAAGCUGUUCCUAAAGA, and siCTR: UGGUUUACAUGUUUUCUGA. RNAiMAX (Invitrogen) was used for the siRNA transfection according to the manufacturer’s instructions.

### Immunopurification and mass spectrometry

Lysates from HeLa cells synchronized by double-thymidine block were prepared in lysis buffer (50 mM Tris-HCl, pH 7.4, 150 mM NaCl, 0.3% Nonidet P-40, 1 mM DTT, and 5 mM EDTA) with protease inhibitor cocktail (Roche) and centrifuged at 14,000 g at 4 °C for 15 min. The supernatants were incubated with anti-FLAG M2 beads at 4 °C for 4 h, followed by washing five times with lysis buffer. Protein complexes were eluted from the beads with 0.1 mg/ml FLAG peptides, collected, and visualized on NuPAGE 4-12% Bis-Tris gel (Invitrogen), followed by silver staining with the Pierce Silver Stain Kit (Thermo Scientific). The protein bands were retrieved and analyzed by liquid chromatography-tandem mass spectrometry with an FDR cut-off less than 1%.

### Chromatin fractionation assay

U2OS cells were synchronized by double-thymidine block and harvested at different time points after release in a fresh medium for analysis of chromatin-binding proteins.^72^ Briefly, 60-mm plates of cells were harvested prior to the addition of 150 μl of cytoskeletal (CSK) buffer (0.5% Triton X-100, 0.1 M NaCl, 10 mM piperazine-N, N‘-bis [2-ethanesulfonic acid], pH 7.0, 300 mM sucrose, 1 mM MgCl_2_, 1 mM EDTA, 2 mM phenylmethylsulfonyl fluoride, 10 mM NaF, 20 mM glycerophosphate, 100 μM Na_3_VO_4_, plus 1× complete protease inhibitor cocktail) and lysis by pipetting up and down. Lysates were placed on ice for 20 min, vortexed every 5 min, and pipetted for five more times. Fifty microliters of lysates were placed in a new tube as the whole-cell extract (WCE) sample, and the remaining lysates were centrifuged at 5000 rpm (~2000 × g) for 5 min prior to transferring supernatants to a new tube as soluble fraction. After washing in 200 μl of CSK buffer by pipetting once, the pellets were resuspended in 100 μl CSK buffer as chromatin fraction. All WCE, soluble fraction, and chromatin fractions were added to the same amount of 2× SDS loading buffer and boiled for 20 min before western blotting.

### iPOND

iPOND assay was performed as described previously.^73^ Briefly, cells were labeled with 10 mM of EdU for 10 min and rapidly harvested and washed with ice-cold PBS. For thymidine chase, cells were washed with PBS to remove EdU and cultured in fresh media with 10 mM thymidine for 30 min, followed by cross-linking with 1% formaldehyde for 10 min, quenching with 250 mM glycine for 5 min, and washing three times with PBS. Cells were then permeabilized with 0.25% Triton X-100 in PBS for 30 min at room temperature, washed with 0.5% BSA in PBS, and resuspended in PBS before click reagents were added in the following order to a final concentration: biotin-azide (100 mM), sodium ascorbate (10 mM), and CuSO_4_ (2 mM) and incubated for 1 h at room temperature. Cells were then washed three times with PBS, resuspended in lysis buffer (1% SDS, 10 mM EDTA, 50 mM Tris-HCl [pH 8.1]), and sonicated with Bioruptor with 30 s on and 30 s off for 30 cycles. After an aliquot was saved for input, PBS was added to dilute the concentration of SDS to 0.5%. Biotinylated DNAs were pulled down using M280 streptavidin Dynabeads (Life Technologies) and boiled for 30 min to reverse cross-links. The beads were then thoroughly washed and the eluted proteins were analyzed by western blotting.

### In situ proximity ligation assay (PLA)

U2OS cells were cultured on chamber slides and pulse-labeled with 10 μM of EdU for 10 min, followed by thymidine chase for 30 min. Cells were then fixed with 4% paraformaldehyde in PBS for 15 min at room temperature, washed with PBS, and permeabilized with 0.3% Triton for 15 min before being washed in PBS three times and then subjected to Click-iT reaction with biotin-azide for 2 h. The PLA reactions between anti-biotin and anti-LSD1 were performed following the Duolink PLA assay instruction. The PLA results were quantified by counting the number of PLA signals per EdU-labeled nuclei in at least 8 cells in each of three independent experiments.^38^

### DNA fiber assay

Cells were successively labeled with 25 μM of IdU (Sigma) and 250 μM of CldU (Sigma) for 30 min and collected for DNA fiber analysis as described.^40^ Briefly, U2OS cells were transfected with siRNAs before labeling. Cells were then collected and suspended in ice-cold PBS to a concentration of 1 × 10^6^ cells/ml. DNA fibers were stretched on salinized coverslips and fixed in methanol/acetic acid (3:1) solution. Combed DNA fibers were denatured in 2.5 M of HCl for 80 min before IdU was detected with a mouse monoclonal antibody (BD44, Becton Dickinson; 1:20 dilution) and a secondary antibody coupled to Alexa 488 (A11006, Invitrogen; 1:50 dilution) and CldU was detected with a rat monoclonal antibody (BU1/75, AbCys; 1:20 dilution) and a secondary antibody coupled to Alexa 546 (A21123, Invitrogen, 1:50 dilution). The coverslips were mounted with ProLong Gold antifade reagent (catalog number P36934; Invitrogen) and imaged by Axioskop inverted microscope with a 63× objective. Experiments were repeated at least twice, and data were acquired with the ImageJ software. Data from independent experiments were pooled, and the distribution of track lengths was plotted as beeswarm plots. A Mann–Whitney *U* test was used to compare the distributions of the track lengths.

### ChIP sequencing

HeLa cells were synchronized in G_1_/S boundary through double-thymidine block and released in fresh medium for 0.5 or 2.5 h before being harvested. Approximately 5 × 10^7^cells were used for each ChIP-seq assay. Chromatin DNAs were precipitated with polyclonal antibodies against LSD1, CDC45, or H3K4me2 and purified with the Qiagen PCR purification kit for deep DNA sequencing by the BGI Corporation (Beijing Genomics Institute). The raw data were quality-checked via Fastx toolkit with default parameters. Sequences were aligned to the unmasked human reference genome (hg38) using Bowtie Version 2^74^ with only one mismatch allowed. MACS Version 2 (Model-based Analysis for ChIP-Seq)^75^ was used for the identification of CDC45 or H3K4me2 peaks with all default settings except *q* < 0.05. Genomic distribution of LSD1, CDC45, and H3K4me2 was visualized in IGV. ORC1 binding sites were considered as licensed origins and H3K27ac/H3K4me2-enriched regions were considered as enhancer-like regions. The origin data from HeLa cells were from the recently published work.^49^ Real-time RT PCR primers were designed for two early origins, Ori #1 and #2, and two late origins, Ori #3 and #4. Ori #1: Forward GCCAGAAGTTGCTGAAAGCG, Reverse TCCTAGCTAACTCTCGGGGG; Ori #2: Forward GCCTGGGTGCTGCTTAATTC, Reverse GTGGAATCCTCGCCTCTACG; Ori #3: Forward AAGGTAGGATAGGGCCTCGG, Reverse ACCGCATGAGTTGCCCTAAA; Ori# 4: Forward CAGCCTCCCCTTACTTACGC, Reverse AAGAGACCAGGGCTCCAGAT.

### Replication sequencing

Repli-seq assays were performed with a modified protocol of Nasent-EdU-IP seq.^45^ Briefly, HeLa cells were transfected with control siRNA or LSD1 siRNA and synchronized to G_1_/S phase by thymidine and aphidicolin double treatments. Cells were cultured with 20 μM of EdU for 10 min after release into S phase by washing out aphidicolin for 0, 3, and 5 h. The cells were collected and lysed immediately in lysis buffer (50 mM Tris-HCl, pH 8.0, 10 mM EDTA, 1 M NaCl, 0.5% SDS, 0.1 mg/ml RNase A, 0.2 mg/ml protease K), and the lysates were incubated at 37 °C for 3 h and then at 55 °C for 8 h before being sonicated for five cycles using Bioruptor with 30 s on and 30 s off for 30 cycles. DNA fragments ranging from 200 to 400 bp were selected with AMPure beads (0.6× to 1.0×) (Beckman, A63881). Biotin was conjugated to EdU-labeled DNAs by Click-iT reaction in buffer containing 15 mM Tris, pH 7.5, 400 μM biotin-azide; 100 μM:500 μM CuSO4 (Sigma, 209198):THPTA (Sigma, 762342), 5 mM freshly prepared sodium ascorbate (Sigma, A4034) at 34 °C for 20 min. DNAs were purified with MinElute PCR purification kit (Qiagen, 28004), end-repaired, dA-tailed, and adapter-ligated before being dissolved in 1× TE buffer and incubated with M280 streptavidin beads (Thermo Fisher, 11205D) in binding/washing buffer containing 5 mM Tris-HCl, pH 7.5, 1 M NaCl, 0.5 mM EDTA, pH 8.0 for 20 min. After rinsing in binding/washing buffer containing 0.5% IGEPAL CA-630 (Sigma, I3021) for six times, recovered DNAs from beads were used for Repli-seq library construction. Deep DNA sequencing was performed by Jiayin biotechnology (Shanghai), and raw reads were preprocessed using fastp^76^ for adapter trimming and low-quality (Phred score <20) base removal. The remaining reads were mapped to Ensembl GRCh37/hg19 reference genome through Bowtie Version 2^74^ with unpaired, discordant and multiple alignments were discarded. The read coverage across genome is calculated in genomic windows of 10 kb and normalized through the quantile method across samples with loess smoothing for noise reduction. Coverage files in bedGraph were visualized with IGV.^77^

### ChIP-seq and Repli-seq

Raw sequence reads for H3K4me2 and H3K27ac were downloaded from ENCODE (https://www.encodeproject.org) for HeLa-S3.^78^ The reads were aligned to the human genome (GRCh38/hg38) using Bowtie Version 2.^74^ ChIP-seq reads density was calculated genome wide with a bin size of 50 bp using deepTools.^79^ Model-based analysis of ChIP-seq (MACS2)^75^ was applied to identify LSD1 peaks with a threshold of *p* < 1e–3. ORC1 ChIP-seq data were downloaded,^49,51^ and Repliscan^80^ was used for calculation and normalization for our Repli-seq data with a bin size of 10 kb.

### RNA-seq

U2OS cells were transfected with control siRNA or LSD1 siRNA for total RNA preparation and sequencing. STAR was used to align clean reads to reference genome, and volcano plots were drawn by R based on differential genes and the color was determined by filtering criteria. Gene Ontology analysis was performed to determine the biological implications of differentially expressed genes, and the significant pathways of the involving genes were analyzed based on KEGG database by ClusterProfiler with defined *p*-value and FDR.

### Synchronization of U2OS cells

For double-thymidine blocks, 1.5 × 10^5^ cells/well were plated in 6-well plates the day before siRNA transfection. After 6 h, the cells were washed and placed overnight in a medium supplemented with 2 mM of thymidine (Sigma) for 14–17 h. After extensive washes, cells were placed in a fresh medium for 10–12 h before the second thymidine block for 14–17 h. Cells were released and harvested at 0, 3, 6, 9, or 12 h for cell cycle analysis. On average, 90% of cells were in G_1_/S at the time of release. To follow cells progressing through S phase, cells were pulse-labeled with EdU for 60 min before harvesting. For thymidine-nocodazole blocks, U2OS cells in log phase were treated for 14–15 h with 2 mM of thymidine and released for 6 h before 250 ng/ml of nocodazole (Sigma) was added to the medium for another 6 h. Cells were released and harvested at 0, 1, 3, 7, or 12 h after extensive washes. For caffeine and UCN-01 treatments, LSD1 siRNA-treated cells were incubated with 5 mM of caffeine (Sigma) dissolved in water at 80 °C at a concentration of 100 mM for 2 h or with 500 nM of UCN-01 (Sigma) dissolved in DMSO at 100 mM for 2 h before a 60-min pulse with 10 μM of EdU for cell cycle analysis by EdU/PI incorporation.

### EdU flow cytometry analysis

U2OS cells were seeded in 6-well plates at 25% confluence the day before transfection with control siRNA or LSD1 siRNA. The medium was changed 6 h later and the cells were harvested 48 h later after 10 μM of EdU labeling for 1 h. The cells were then washed in PBS containing 1% BSA, and fixed with 4% paraformaldehyde for 15 min at room temperature in dark, followed by washing with PBS/1% BSA, permeabilizing with 0.2% saponin in PBS/1% BSA for 15 min, and click-labeling. Click reaction cocktail was mixed according to the instructions of Click-iT EdU Flow Cytometry Assay Kit, and 0.5 ml was added to each tube that was well-mixed. The reaction mixture was incubated for 30 min at room temperature in dark. Cells were then washed with 0.2% saponin in 1% PBS/1% BSA before each sample was added with 500 μl of saponin buffer supplemented with ribonuclease A and PI for RNA removal and DNA content staining, respectively. Cells were filtered into tubes and incubated at room temperature for 1 h before being analyzed on a flow cytometer.

### Cell viability/proliferation assay

For cell proliferation assays, U2OS cells were transfected with control siRNA or LSD1 siRNA and seeded into 96-well plates. The cells were then treated with 100 nM, 500 nM, or 1 μM of CPT, 1 mM, 5 mM, 10 mM of HU, or 5, 30, 60 μM of APH for 24 h. On the day of harvesting, the cells were cultured in an equal volume of fresh medium containing 10% alamarBlue and incubated at 37 °C for 6 h before cell viability measurement by the absorbance of the converted dye at wavelengths 570 and 630 nm.^81^

### Statistical analysis

Results are presented as mean ± SD unless otherwise noted. SPSS version 18.0 was used for statistical analysis, and Student’s *t*-test was conducted to determine the significance. A two-tailed Mann–Whitney *U* test was used to compare the population distribution between two groups.

## Supplementary information

Supplementary Material

Table S1

Table S2

## Data Availability

All data needed to evaluate the conclusions in the paper are present in the paper and/or the Supplementary Materials. The high throughput raw and processed data have been deposited in GEO (https://www.ncbi.nlm.nih.gov/geo/query/acc.cgi?acc=GSE157471).
