# Correction: LSD1 is required for euchromatic origin firing and replication timing

**DOI:** 10.1038/s41392-025-02203-0

**Published:** 2025-04-03

**Authors:** Yue Wang, Yunchao Huang, Edith Cheng, Xinhua Liu, Yu Zhang, Jianguo Yang, Jordan T. F. Young, Grant W. Brown, Xiaohan Yang, Yongfeng Shang

**Affiliations:** 1 Department of Biochemistry and Molecular Biology, School of Basic Medical Sciences, Peking University Health Science Center, Beijing, 100191 China; 2 Department of Biochemistry and Molecular Biology, School of Basic Medical Sciences, Hangzhou Normal University, Hangzhou, 311121 China; 3 Department of Biochemistry and Molecular Biology, School of Basic Medical Sciences, Capital Medical University, Beijing, 100069 China; 4 Department of Biochemistry and Donnelly Centre, University of Toronto, Toronto, ON M5S 1A8 Canada; 5 Lunenfeld-Tanenbaum Research Institute, Mount Sinai Hospital, 600 University Ave., Toronto, ON M5G 1×5 Canada

Correction to: *Signal Transduction and Targeted Therapy* 10.1038/s41392-022-00927-x, published online 13 April 2022

Following online publication of article 1, unintentional errors were identified in Figure 1d and supplementary figure S2b. These have been corrected, and the updated figures are provided below. Importantly, these corrections do not alter the article’s key findings.

During figure assembly, the blots of HA-MCM7 were duplicated in the last two panels in Fig. 1d. The correct results should be as shown below.

Incorrect Figure 1d
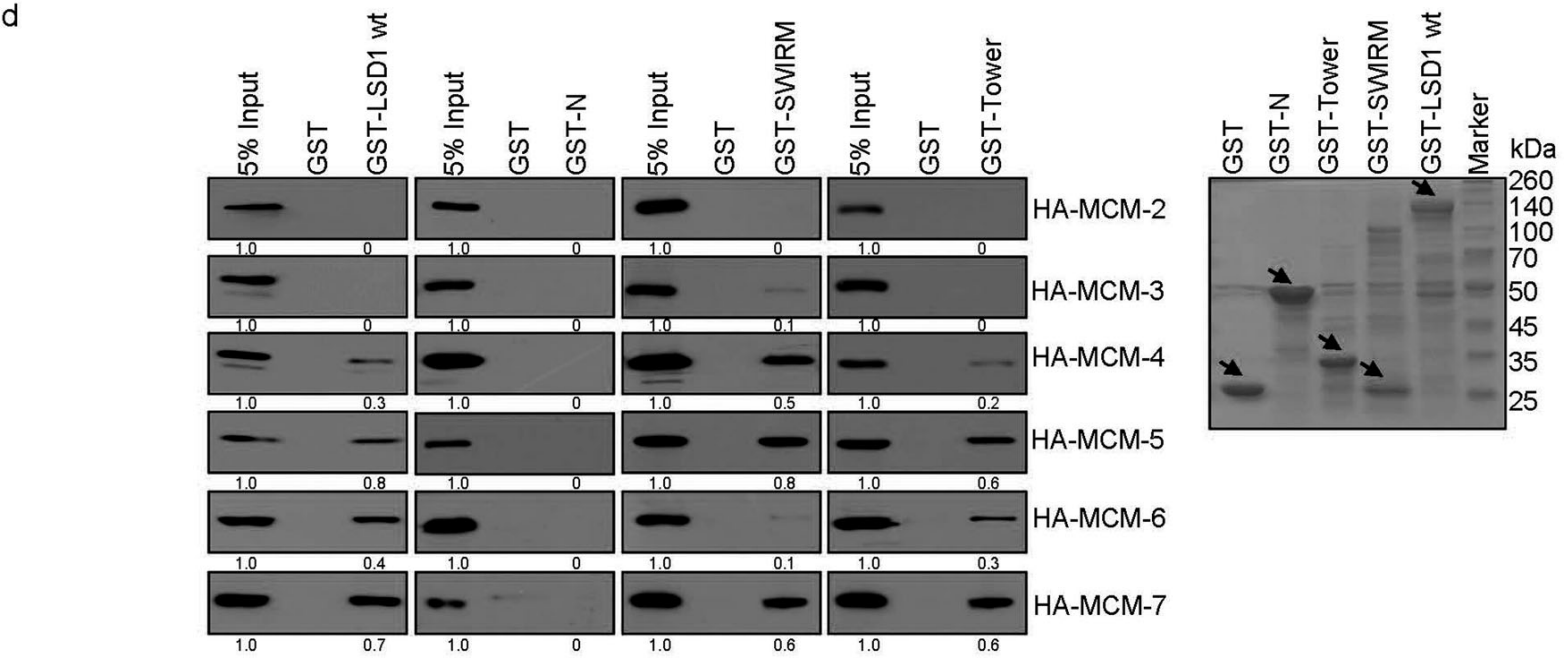


Corrected Figure 1d
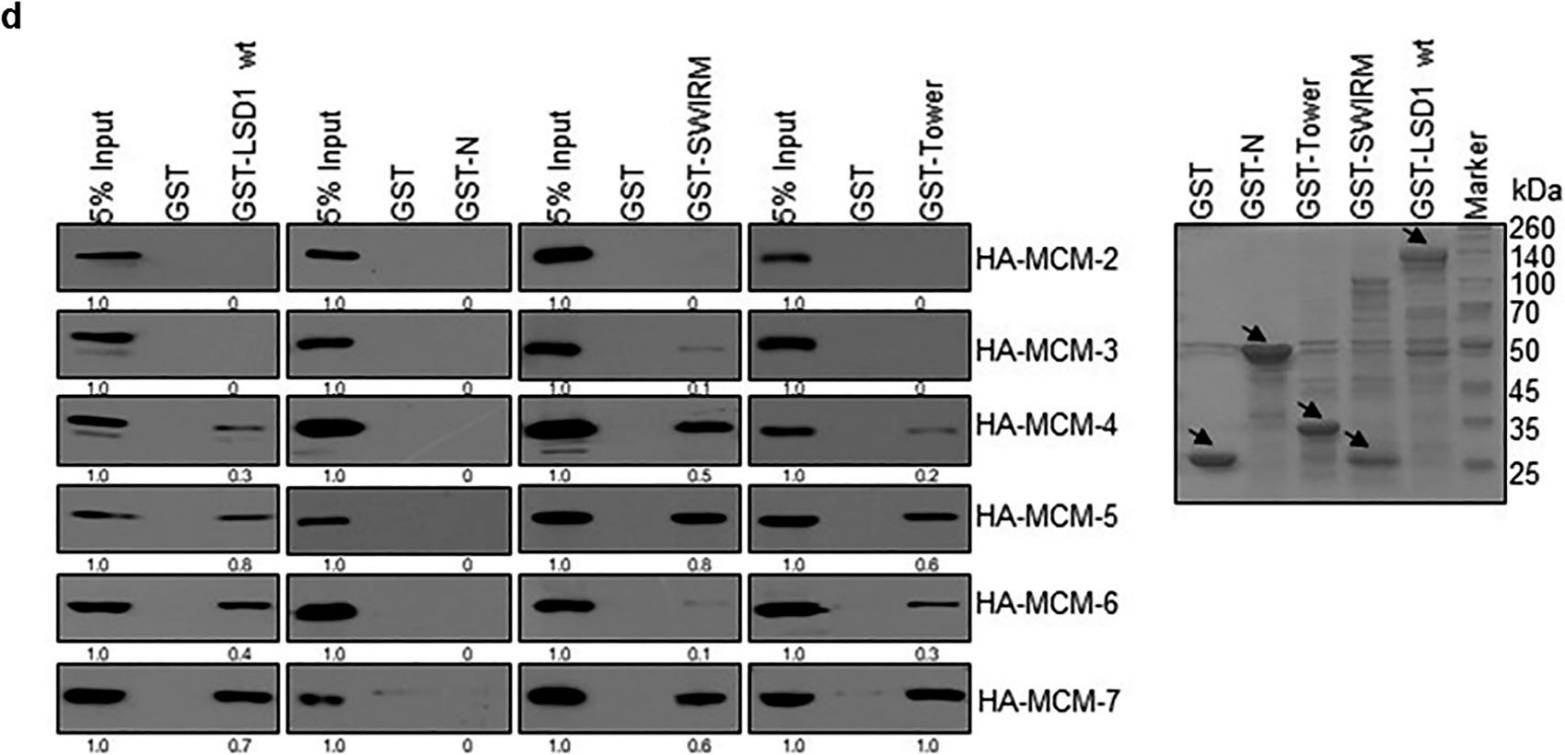


Fig. 1d GST pull-down assays with GST-LSD1, GST-N (1–171 aa), GST-SWIRM (171–271 aa), and GST-Tower (416–521 aa) and in vitro transcribed/translated MCM2–7.

During the preparation of Supplementary Fig. S2b, representative images for siLSD1#1, siLSD1#2, siPCNA, and siFANCM duplicated by mistake the representative images for siCTR (CPT), siLSD1#1 (CPT), siCTR (APH), and siLSD1#1 (HU), respectively, in Fig. S3a. The correct results should be as shown below.

Incorrect Figure S2b:
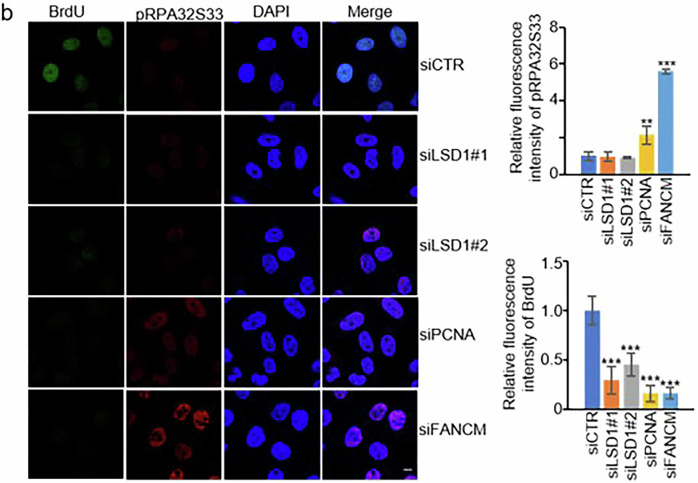


Correct Figure S2b
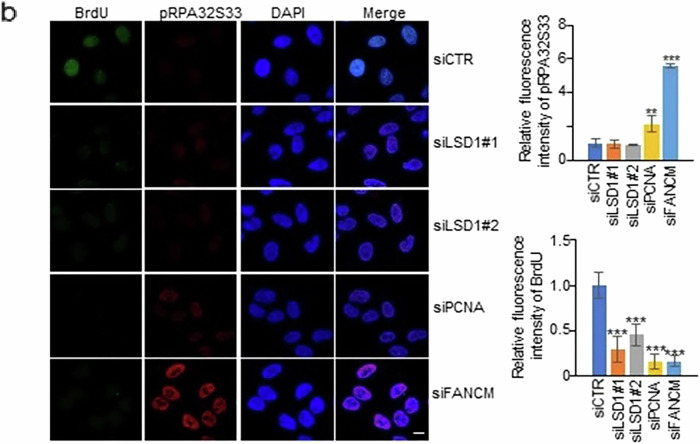


Fig. S2b. HeLa cells were treated with the indicated siRNAs and pulse-labeled with BrdU for 30 min before being harvested. pRPA32S33 and BrdU were stained. FANCM was a positive control. Scale bar, 10 μm.

The original article has been corrected.

