# Supplementary material for: LSD1 is required for euchromatic origin firing and replication timing
Source: Signal Transduct Target Ther. 2022 Apr 13;7:102. doi: 10.1038/s41392-022-00927-x (PMC9005705; doi:10.1038/s41392-022-00927-x)
Supplement: Supplementary file 1 — Supplementary Material [file 41392_2022_927_MOESM1_ESM.docx]

Supplementary Materials for

LSD1 Is Required for Euchromatic Origin Firing and Replication Timing

Yue Wang^1,2,3^, Yunchao Huang^1^, Edith Cheng^4^, Xinhua Liu^2^, Yu Zhang^1^, Jianguo Yang^1^, Jordan T. F. Young^5^, Grant W. Brown^4^, Xiaohan Yang^1,5,6^, and Yongfeng Shang^1,2,3,6^

Corresponding author. Email: Xiaohan Yang (xiaohanyang@hsc.pku.edu.cn)

Yongfeng Shang (yshang@hsc.pku.edu.cn)

**This PDF file includes:**

Figure. S1 to S6

Figure S1


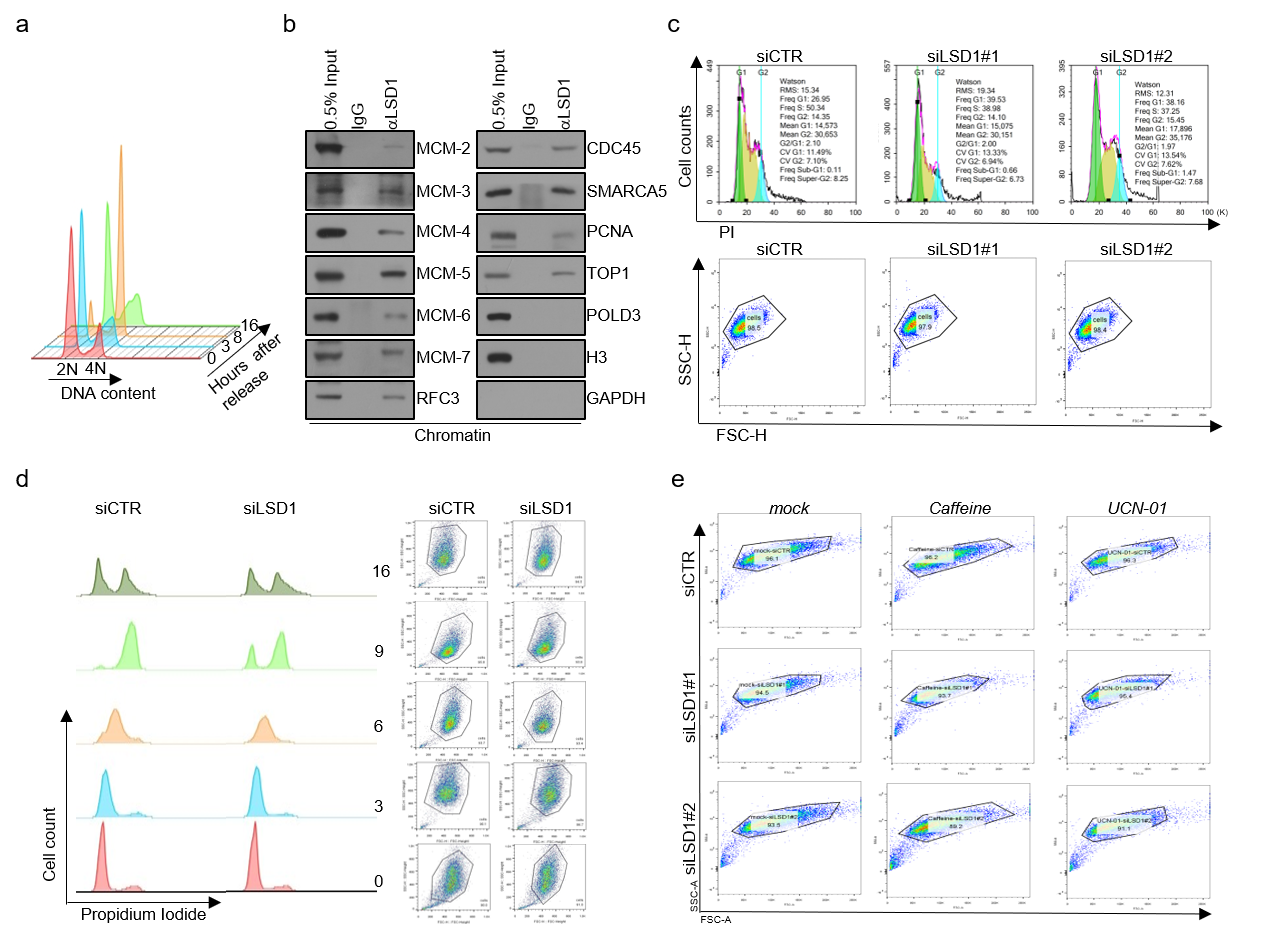


**Figure. S1. a** Cell cycle plots at the indicated times after release for Fig. 1a. **b** LSD1 interacts with MCM2-7, CDC45, RFC3, SMARCA5, PCNA and TOP1 on chromatin. Immunoprecipitation of released chromatin proteins in S-phase HeLa cells with the antibody against LSD1, followed by immunoblotting with antibodies against the indicated proteins. GAPDH was used as a negative control. **c** Cell cycle plot (upper panel) and gate setting (lower panel) for Fig. 3a. The percentage of cells is labeled in the gate. **d** Representative full cell cycle plots of LSD1-deficient U2OS cells (left panel) for Fig. 3c. Corresponding gates setting were shown in the right panel. **e** Gate setting for Fig. 3d. The percentage of cells was shown in the gate.

Figure S2


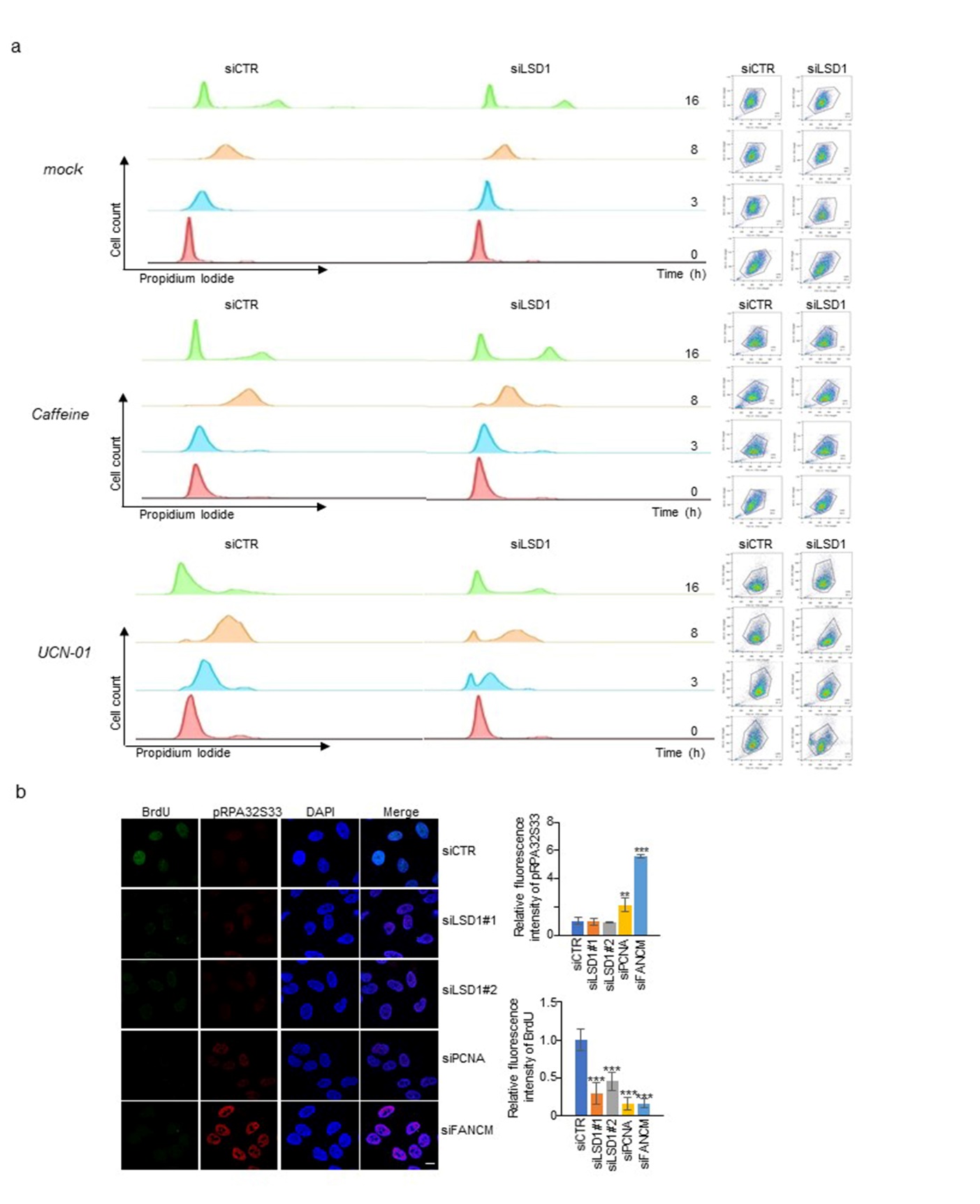


**Figure. S2. a** Cell cycle plots for LSD1-deficient U2OS cells challenged with Caffeine or UCN-01(left panel). Corresponding gates setting are shown (right panel). The X-axis represents FSC-H, and the Y-axis represents SSC-H. **b** HeLa cells were treated with the indicated siRNAs and pulse-labeled with BrdU for 30 min before being harvested. pRPA32S33 and BrdU were stained. FANCM was a positive control. Scale bar, 10 μm. Quantification of immunostaining intensity of pRPA32S33 and BrdU (right). *P*-values were calculated by a two-tailed student’s *t*-test.


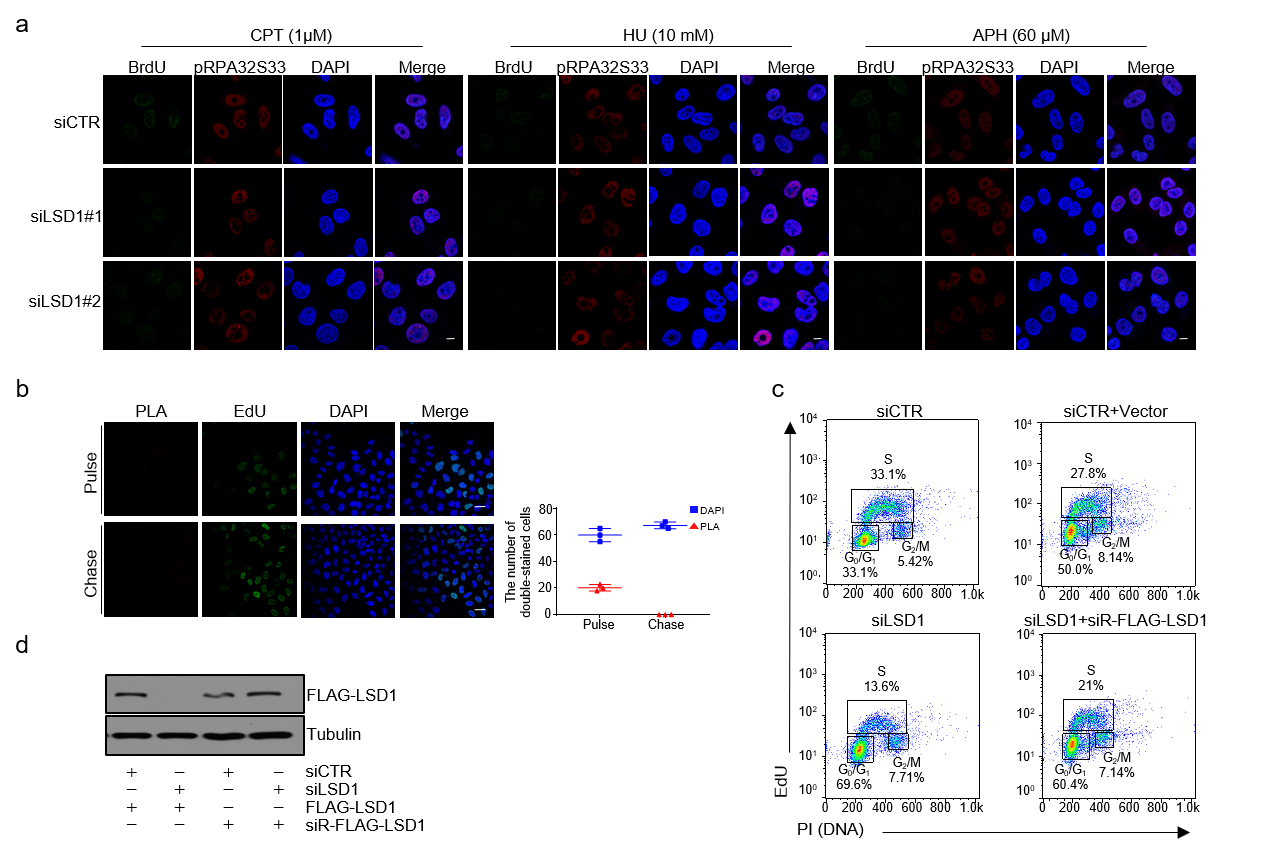
 Figure S3

**Figure. S3. a** U2OS cells transfected with control or LSD1 siRNAs were treated with CPT, HU or APH, and pulse-labeled with BrdU for 30 min before being harvested. Immunostaining of pRPA32S33 and BrdU was performed. Scale bar, 10 μm. **b** U2OS cells were pulse-labeled with EdU followed by thymidine chase for *in situ* PLA assays. Scale bar, 25 μm. The number of double-stained cells was counted (right panel). Each bar represents mean ± S.D. for three independent experiments. **c** LSD1-deficient LSD1 cells were transfected with FLAG-LSD1/siRNA resistant FLAG-LSD1 vectors and subjected to EdU incorporation analysis. The percentage of cells in each phase of the cell cycle was determined by dual PI/EdU flow cytometry. **d** LSD1-deficient LSD1 cells were transfected with FLAG-LSD1/siRNA resistant FLAG-LSD1 vectors. The expression of LSD1 was detected by western blotting.


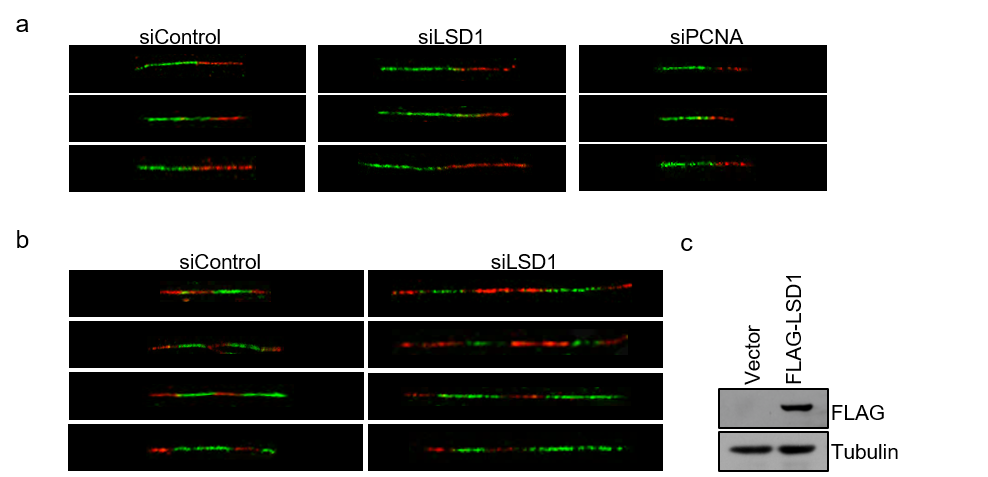
Figure S4

**Figure. S4.** **a-b** Representative DNA fibers for U2OS cells treated with indicated siRNAs for Fig. 4b and Fig. 4c, respectively. **c** HeLa cells were transfected with empty or FLAG-LSD1 vector for western blotting with antibodies against FLAG or Tubulin.


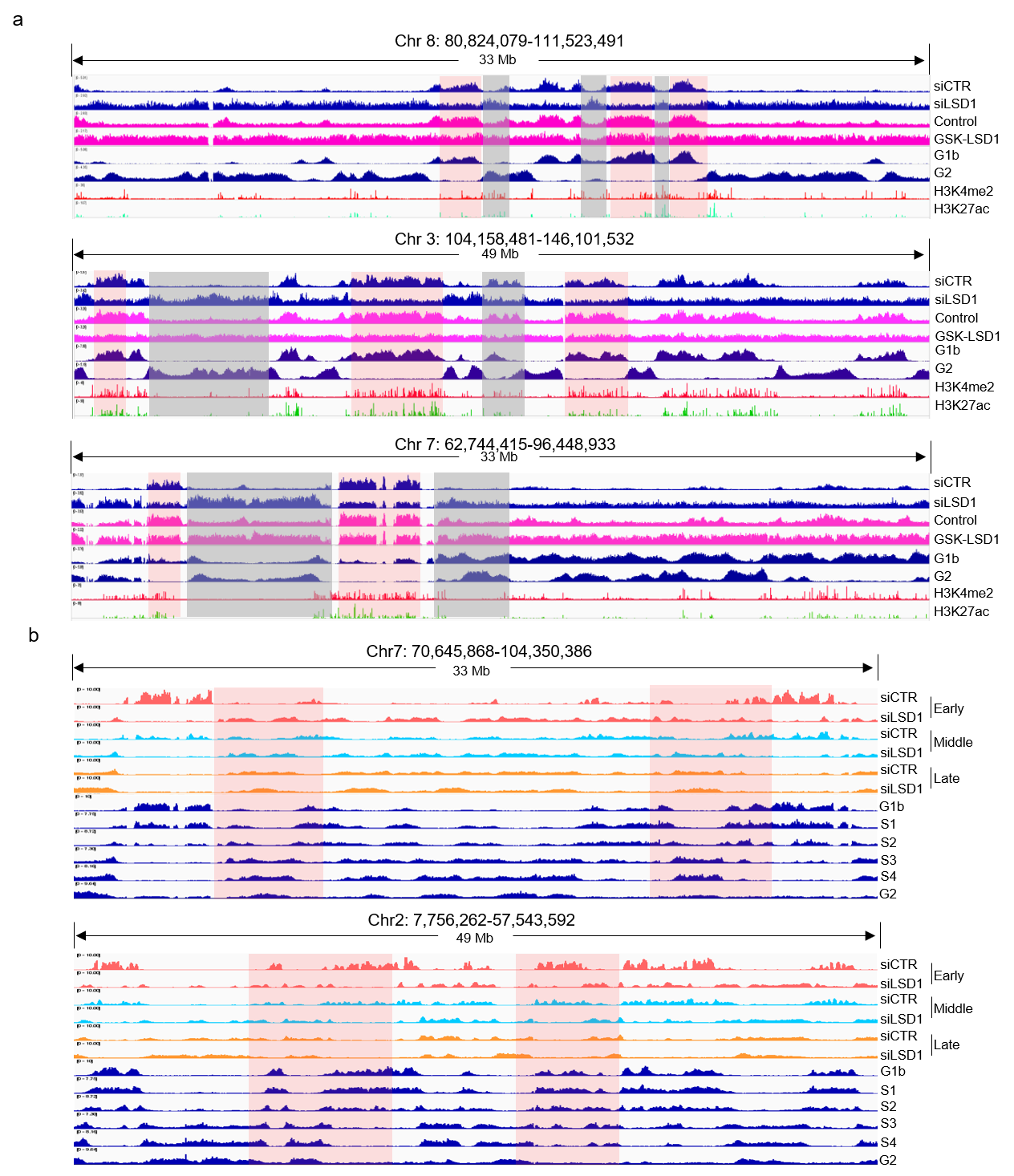
Figure S5

**Figure. S5. a** Representative regions of chromosome 8 spanning 33 Mb, chromosome 3 spanning 49 Mb and chromosome 7 spanning 33 Mb are illustrated. Regions with decreased Repli-seq reads density after LSD1-depletion/inhibition is shaded in pink, and regions with increased reads density shaded in grey. Replication profiles from public data representing early (G1b) and late (G2) S phases (below) and the distribution of H3K4me2 and H3K27ac (bottom) are shown. **b** Different time points of synchronized LSD1-depleted HeLa cells were analyzed by Repli-seq. Replication profiles from public data representing early (G1b and S1), middle (S2 and S3), and late (S4 and G2) S phases (dark blue) are shown below. Representative regions of chromosome 7 spanning 33 Mb and chromosome 2 spanning 49 Mb are illustrated.


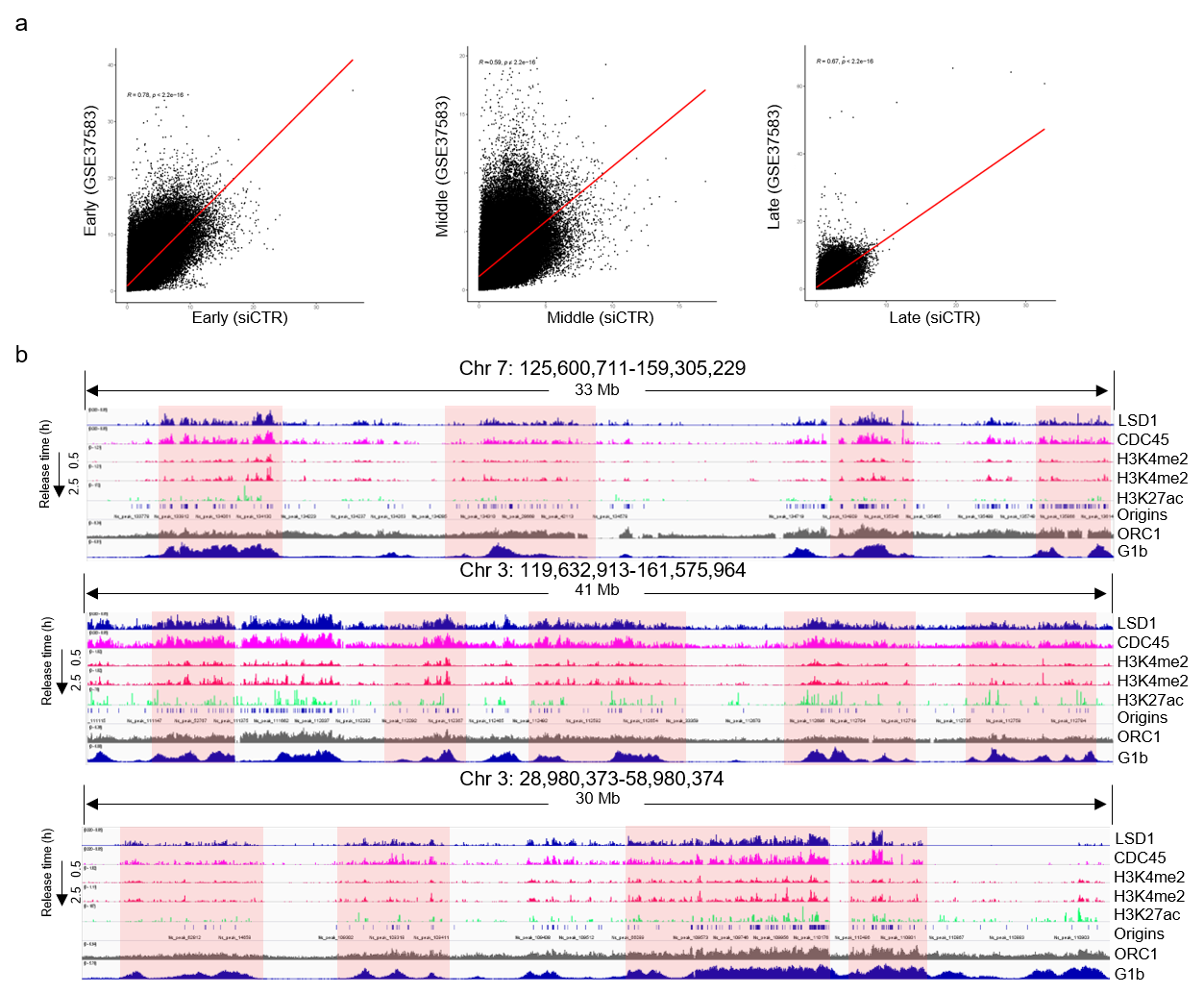
Figure S6

**Figure S6. a** Correlation analysis of genome-wide Repli-seq signal between our control groups and the published dataset (GSE37583) at different time points. The whole genome was segregated with the bin size of 10 kb and the Repli-seq signal within each bin was represented as the RPGC (reads per genome coverage) normalized raw reads count. *r*, Pearson’s correlation coefficient. **b** Representative regions in chromosome 3 and chromosome 7 are shown. Origins data in HeLa cells were from published data. Public Repli-seq data of early S phase (G1b) is shown below. Co-occurrence of LSD1, CDC45 and H3K4me2 on early-replication regions are shaded.
